# Federated Learning in Edge Computing: Vulnerabilities, Attacks, and Defenses—A Survey

**DOI:** 10.3390/s26041275

**Published:** 2026-02-15

**Authors:** Sahar Alhawas, Murad A. Rassam

**Affiliations:** Department of Information Technology, College of Computer, Qassim University, Buraydah 52571, Saudi Arabia

**Keywords:** federated learning, edge computing, security, privacy, vulnerabilities, adversarial attacks

## Abstract

Federated Learning (FL), a distributed machine learning framework, enables collaborative model training across multiple devices without sharing raw data, thereby preserving privacy and reducing communication costs. When combined with Edge Computing (EC), FL brings computations closer to data sources, enabling low-latency, real-time decision-making in resource-constrained environments. However, this decentralization introduces several vulnerabilities, including data poisoning, backdoor attacks, inference leaks, and Byzantine behaviors, which are worsened by the heterogeneity of edge devices and their intermittent connectivity. This survey presents a comprehensive review of the intersection of FL and EC, focusing on vulnerabilities, attack vectors, and defense mechanisms. We analyze existing methods for robust aggregation, anomaly detection, differential privacy, and secure aggregation, with a focus on their feasibility within edge environments. Additionally, we identify open research challenges, such as scalability, resilience to heterogeneity, and energy-efficient defenses, and provide insights into the evolving landscape of FL in edge computing. This review aims to inform future research on enhancing the security, privacy, and efficiency of FL systems deployed in real-world edge environments.

## 1. Introduction

The acceleration in data generation from IoT devices, smartphones, and edge systems has only heightened the urgency to deploy privacy-sensitive distributed learning models [1]. Federated Learning (FL) has become a paradigm-shifting approach in the scope of distributed machine learning that is sensitive to the privacy issues of the data and seeks to implement collaborative model training involving many decentralized clients connected within a network [2,3], which is essential when the data of the devices is sensitive, like health measurements, user behavior, sensor data, etc. [4,5]. Edge computing complements FL by decentralizing computation and bringing it closer to the data source. The synergy between FL edge computing and smart decision-making is a key driver of smart decision-making, particularly in low-latency environments such as industrial IoT [1], automotive systems [2], in-home health monitoring [3], and innovative healthcare [6]. The rapid increase in data generated by smartphones, sensors, and embedded systems has only underscored the need for privacy-friendly distributed learning. FL is best suited because it enables devices to contribute to a common model without exposing raw data on health status, user behavior, or physical environments.

There are unique security and privacy threats associated with the use of FL in edge environments. Geographically distributed edge devices with resource constraints are vulnerable to adversaries who can exploit training or access information [7]. Poisoning attacks include corrupting local updates before aggregation [8], backdoor injections [9], implanting trigger-conditioned behavior in an otherwise unchanged validation performance, and inference or gradient-based attacks, which are concrete and recover sensitive training data using shared updates [10,11]. These vulnerabilities are primarily attributable to the absence of centralized control, untrusted device hardware, and intermittent connectivity of edge devices, all of which increase the susceptibility of FL systems to attacks [12].

Although several defense mechanisms exist, such as strong aggregation [13], differential privacy, and anomaly detection [14], they tend to be less effective in resource-constrained edge settings. Most defense mechanisms operate in cloud environments or idealized conditions that are not always aligned with the constraints of edge devices. These threats are compounded at the edge, where devices and communication infrastructure are not uniformly hardened. Moreover, device processing, memory, and energy resources are limited at the edge, making it difficult to rely on heavy cryptographic or statistical security [15]. In contrast, mechanisms such as strong aggregation [13], differential privacy, and anomaly detection have been proposed [15], many of them assume cloud-like resources or IID data and, therefore, become challenging within the constraints of edge devices.

However, edge deployment exacerbates legacy machine learning problems, including non-I.I.D. data distribution, heterogeneous hardware/software stacks, intermittent connectivity, and small-scale computation and power requirements [16]. Such conditions make convergence slower and training less stable and increase the probability and effects of successful attacks, especially when devices are in semi-trusted or untrusted environments.

To address these issues, recent research has focused on understanding the architectural foundations of FL, identifying and classifying adversarial threats, and developing robust defense mechanisms for a heterogeneous, resource-constrained edge environment. Numerous surveys in the literature aim to describe the FL landscape—some focus on taxonomy and frameworks, others on security and privacy. Despite the breadth of existing surveys on federated learning, relatively few studies focus on edge computing environments while analyzing adversarial attacks and their corresponding defense mechanisms. Table 1 presents an overview of previous survey studies on federated learning and edge computing.

### 1.1. Methodology

This survey employs a qualitative, structured literature review methodology. Rather than merely summarizing existing studies, the survey categorizes, contrasts, and critically analyzes prior work on the security and adversarial robustness of federated learning systems deployed in edge computing environments. To ensure the survey’s credibility and relevance, we focused on research published in well-known academic journals and on widely used preprint platforms. The reviewed studies were obtained from sources including IEEE Xplore, ACM Digital Library, SpringerLink, Elsevier ScienceDirect, MDPI, Google Scholar, and arXiv. Most of the selected papers were published between 2019 and 2025, although a small number of earlier works were included when they were essential for background or context.

The following were the inclusion criteria:

- Papers concerning federated learning security or privacy;
- Direct attention to edge computing, IoT, or resource-constrained environments;
- Attack, defense, or system-level constraint discussion.

The exclusion criteria were:

- Papers considering cloud-based FL only, and none about the edges;
- Purely theoretical cryptographic schemes are not mentioned to be implemented;
- Research confined to machine learning in centralized or non-federated paradigms. Following an initial screening of titles and abstracts, selected papers were reviewed and categorized according to threat models, attack objectives, defense mechanisms, and edge feasibility considerations.

### 1.2. Contributions

This survey differs from previous studies in four respects. *First*, it focuses on federated learning in edge computing and examines how decentralization, heterogeneous hardware, intermittent connectivity, and energy constraints reshape the threat environment and the capabilities of defenses. *Second*, it proposes a threat-based taxonomy that categorizes attacks based on adversarial goals: integrity, privacy, availability, and communication, and bases each of the categories on the conditions of realistic deployments of edges. *Lastly*, it uncovers gaps in the existing literature, such as discrepancies between proposed threat models and real-world edge constraints, and the lack of standardized benchmarks for assessing security in edge FL.

These observations indicate the absence of a unified survey examining vulnerabilities, attacks, and defenses in federated learning under edge constraints.

Meanwhile, this environment of decentralization and resource constraint also poses a significant threat to security. Edge clients are heterogeneous in both software and hardware; their data are highly non-IID, connections are lossy, and membership is dynamic, which deteriorates convergence and increases the attack surface [19,23]. Adversaries may corrupt training by introducing poisoned local updates [7] or implanting covert backdoors that cause malicious behavior at inference [9] or by introducing Byzantine updates that degrade availability. Even legitimate but curious observers can build inference and gradient-leakage attacks that reconstruct or infer sensitive training information using shared updates [7]. The risks are higher at the edge, as devices and edge servers often lack substantial, uniform hardening. Energy and compute constraints preclude heavyweight cryptography or repeated secure rounds, and non-IID statistics can undermine the usefulness of classical robust aggregation and anomaly detection [24]. The overall effect is a paradigm of the powerful but vulnerable: FL + EC can provide privacy and responsiveness, but because it is decentralized and limited, it is more challenging to design and validate its defenses.

This survey is motivated by the need to compile a focused perspective on vulnerabilities, attacks, and defenses in FL in edge settings, based on deployments and architectures that span cross-device and cross-silo situations, hierarchical (end-edge-cloud) aggregation, and decentralized variants. We also position new directions such as decentralized, blockchain-powered coordination, moving-target defenses against communication threats, and personalized FL, and emphasize evaluations and benchmarks that account for edge limitations rather than idealized lab environments.

This paper presents a structured survey of Federated Learning (FL) on edge computing, focusing on vulnerabilities, attacks, and defenses under realistic edge conditions. We briefly overview the basics of FL and then situate it in the wider context of edge computing. Extending this perspective, we discuss defense mechanisms and evaluate their feasibility on constrained edge devices. It then reviews the literature of currently available studies on FL with reference to edge computing. Two key themes:

- Federated Learning in Edge Computing.
- Security of Federated Learning at the Edge.

Four critical themes have been identified where the review is structured:

1. Federated learning architectures and types.
2. Situation of use at the edge.
3. Adversarial threats and attacks.
4. Defense techniques.

A comparative analysis is also conducted to identify research gaps, especially in attack-defense matchups and benchmark inconsistencies.

**Figure 1:**
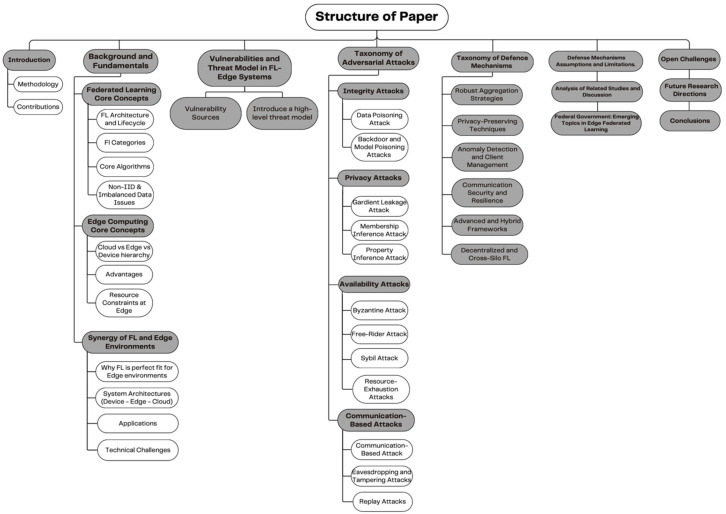
Structure of Paper.

Figure 1 shows the survey structure. Our starting point is the review of FL foundations and types, as well as EC deployment models and system constraints, and defining the architectural context of edge-resident learning. Next, we present a taxonomy of threats to FL at the edge: poisoning, backdoors, inference/leakage, and Byzantine behaviors—and discuss how each is compounded by heterogeneity, non-IID data, compression, and intermittent connectivity. We then present a taxonomy of defense mechanisms, with specific reference to their footprints in the computational memory and communication of devices and edge servers. Lastly, we identify open problems and evaluation gaps, including energy-aware defenses, scalable robustness under severe heterogeneity, leakage under compressed updates, edge-realistic benchmarks, and state-of-the-art research directions for constructing FL systems that are effective and defensible at the edge. Figure 1 presents the structure of this survey.

## 2. Background and Fundamentals

**Figure 2:**
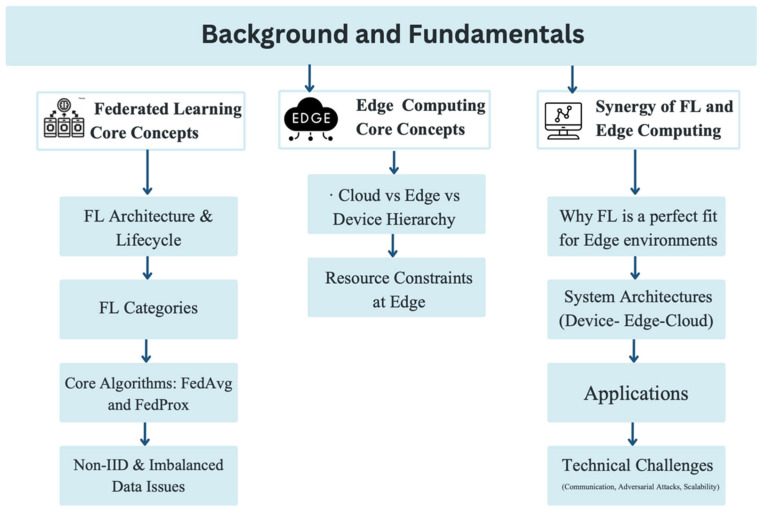
Background and Fundamentals.

This section provides an overview of key concepts, offering a deeper understanding of the fundamentals of Federated Learning and Edge Computing and their synergy, as illustrated in Figure 2.

### 2.1. Federated Learning Core Concepts

Federated Learning (FL) is a distributed machine learning framework that enables multiple clients to jointly train models without exchanging raw data. Instead, local devices calculate model updates that are centralized by a single server or implemented in decentralized protocols, without compromising data privacy and utilizing different datasets [17,19]. This paradigm addresses the issues of data control, security [40], and bandwidth [41] that are inherent to traditional centralized learning methods.

**Figure 3:**
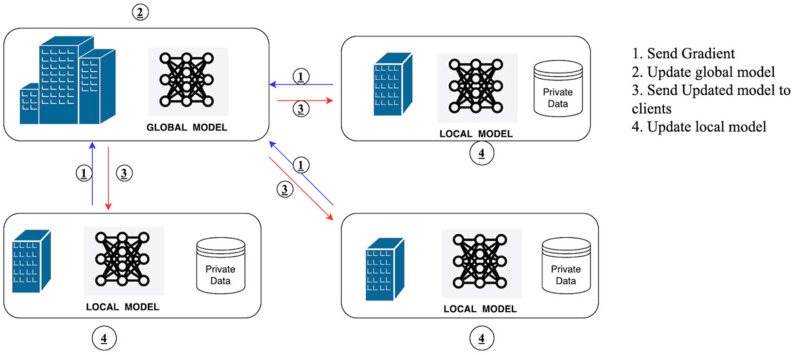
Federated Learning Core Components.

Its basic structure comprises three elements: clients (data owners), an orchestration mechanism (centralized or decentralized), and an aggregation algorithm [19] illustrated in Figure 3. Clients use locally trained models and exchange gradients or parameters rather than sensitive data [42].

Aggregation schemes, such as FedAvg [19], matched averaging [43], or adaptive sampling [16], are used to assemble updates into a global model. Recent developments also consider hierarchical and cross-silo structures to enhance scalability and manage heterogeneity [44].

The primary benefits of FL are privacy, reduced communication overhead, and the ability to leverage heterogeneous data sources in various fields, including healthcare [6], automotive systems [2], and energy forecasting [11]. To elaborate, FL has enabled real-time health tracking at the edge without sending patients’ sensitive health records [3].

Nevertheless, these advantages come with some difficulties. IID data distributions, variability in client resources, and communication bottlenecks [45] are the most common factors that hamper model performance [46]. In addition to that,

FL is susceptible to adversarial attacks such as poisoning, backdoor [9], and inference attacks [7]. Byzantine-resilient aggregation has been proposed in [14,47], and privacy-preserving, backdoor-specific defenses, such as FLIP, have been proposed to enhance robustness [48].

In brief, FL reinvents collaborative intelligence through three dimensions: model accuracy, efficiency, and privacy. Its original ideas, such as client-side training, secure aggregation, and distributed orchestration, serve as a foundation for advancing edge intelligence, cross-domain applications, and privacy-conscious AI [19].

#### 2.1.1. Federated Learning Architecture and Lifecycle

The distributed architecture of Federated Learning (FL) allows the simultaneous training of a machine learning model across multiple devices (clients), with data stored locally and kept private. The FL architecture consists of a server and multiple client units. All clients train their own models on their own data and upload model updates (typically gradients or weights) to the central server [32]. These updates are then combined by the server, usually via weighted averaging, and the global model is updated [49]. After updating the global model, it is retransmitted to the clients for further local training, and the process repeats until convergence is achieved [2]. This architecture enables training a shared model without violating the privacy of the information stored on each client [14].

Beyond its basic workflow, FL systems can be generally characterized into three types of architectures:

- Centralized
- Decentralized
- Hierarchical

**Figure 4:**
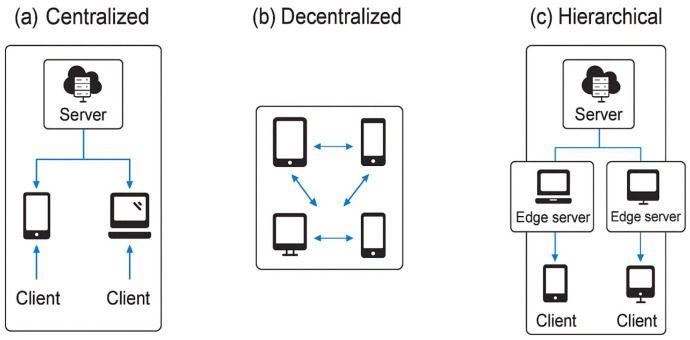
Federated Learning Architectures: (**a**) Centralized, (**b**) Decentralized, and (**c**) Hierarchical.

With different communication patterns, coordination mechanisms, and trust assumptions, illustrated in Figure 4.

Centralized FL is the most common architecture, in which a centralized server aggregates locally trained models from distributed devices. The server broadcasts a global model to the clients, which then train locally on their private training data and send  their updates back to the server for aggregation. Without loss of generality, while the centralized FL facilitates coordination and optimization, it also introduces a single point of failure, the risk of model inversion, and even the threat of denial-of-service [29]. These issues make centralized FL the best fit for scenarios where the central server is highly trusted and secure.

In Decentralized FL, the centralized aggregator can be removed. Instead, model updates are shared among participants using gossip learning or a blockchain-based ledger, for instance. This increases fault tolerance and eliminates the communication bottleneck but introduces synchronization and consistency problems. For example, decentralized FL architectures require sophisticated trust management protocols to address adversaries and malicious devices that could submit poisoned updates [18,50]. Decentralized FL can even achieve performance comparable to centralized FL when properly coordinated, particularly in edge computing, where nodes are sporadically connected [51].

**Hierarchical FL** combines both paradigms by structuring the learning process in a hierarchical manner. In such scenarios, edge devices report to an intermediate node (e.g., a local gateway or regional server), which aggregates the initial model and then transmits the results to a central aggregator [52]. Such architecture is scalable and lowers latency; therefore, it is promising for tiered edge network deployments, including smart city and industrial IoT systems [53]. Moreover, HFL also supports localized personalization, allowing intermediate aggregators to store a portion of the learning specific to their region, which can be generalized more effectively across  different clients [54].

FL has several life cycle stages. The model is first initialized on the central server and then distributed to the participating clients [55]. Local training is performed on the client’s dataset, and the model parameters are updated using it. Thereafter, every client transmits its model updates (but not the data itself) to the central server [14]. These updates are then compiled by the server, which typically employs methods such as FedAvg (Federated Averaging), and the global model is updated accordingly [55]. The new global model is then returned to the clients, and the process repeats. This process is repeated until the model achieves a predetermined level of accuracy or convergence.

#### 2.1.2. Federated Learning Categories

Federated learning can be divided into three main categories based on the distribution of data across clients and the training method.

**Horizontal Federated Learning**: This type is applied when clients’ datasets have identical feature spaces but dissimilar data samples. In horizontal FL, clients have varying datasets, but the features across datasets are equal. This is the simplest type of FL, appropriate for situations where the data can be partitioned into non-overlapping, identically structured partitions [29]. For example, different hospitals may contain different patient information, yet they can share similar clinical features (such as age, gender, and medical conditions) to train a shared health model.

**Vertical Federated Learning**: Vertical FL, on the other hand, is used when clients have similar data (i.e., information about the same entities or users) but different feature sets. This situation arises when data about the same set of individuals exists in multiple forms across two or more organizations [56]. An example would be a bank potentially holding users’ financial data, and an insurance company potentially holding users’ medical history [57]. Vertical FL allows the two organizations to jointly train a universal model without the transmission of sensitive information [58].

**Transfer Federated Learning**: Transfer FL is a hybrid of FL and transfer learning, in which the model trained on one set of clients (or client group) is adapted and transferred to another set of clients with limited data. This type is convenient in situations where some clients have a lot of data and others are data-poor [13]. Performance on larger, data-limited domains can be improved by transferring knowledge from a well-trained model trained on smaller, data-limited domains. The method is beneficial in IoT and medical applications where data availability can be inconsistent [40].

#### 2.1.3. Core Aggregation Algorithms: FedAvg and FedProx

The fundamental federated learning algorithms govern how model updates are aggregated. FedAvg (Federated Averaging) and FedProx (Federated Proximal) are two notable algorithms.

- *Federated Averaging (FedAvg):* FedAvg is the most popular FL algorithm. Under this model, once local training is complete, clients submit their model updates to the central server [55]. The server then averages them to generate a new global model. Weighted averaging is usually performed on the model update for each client. This approach works when the data across clients are IID (Independent and Identically Distributed), i.e., the samples are evenly distributed [20]. Nonetheless, FedAvg can be problematic when client data is highly heterogeneous, as is frequently the case [43,59].
- *FedProx (Federated Proximal):* FedProx was developed to overcome the shortcomings of FedAvg when the data is not IID. The algorithm introduces a proximal term to the local training objective, helping align local updates with the global model [60]. This change helps minimize the adverse impact of data heterogeneity by promoting client focus on improving global models while addressing local data variations [58]. FedProx is particularly applicable in situations where the distributions of client data are quite different, a common phenomenon in real-world settings such as healthcare and IoT networks [61]. Table 2 summarizes the most well-known federated learning aggregation algorithms.

#### 2.1.4. Non-IID and Imbalanced Data Issues

Addressing non-IID (non-independent and identically distributed) data is a primary challenge in federated learning for supervised classification. In traditional machine learning, the data is assumed to be IID: each data point is drawn from the same distribution and is independent of other data points [64]. In FL, however, the data of each client cannot be identical to that of other clients, so it is not IID. The problem here is that clients can access different representations of the data depending on their surroundings, usage habits, or even their domain knowledge. Indicatively, each hospital in a healthcare FL model may have datasets with varying demographics, medical conditions, or data quality, resulting in data that are not identically distributed [17].

The non-IID nature of the data poses several problems, especially in federated classification. This can reduce model performance, as updates from clients with different data distributions may not generalize effectively [14]. Additionally, clients whose data is more representative may take control of the aggregation, resulting in an underperforming client with a small or skewed dataset. To overcome these issues, many methods, including weighted averaging of updates based on client data distributions or adding model specialization, such as FedProx, have been proposed to bring local models closer to the global model [13].

Another related problem is imbalanced data, where some categories are not represented equally across clients. Consider, for example, a financial fraud detection system: fraudulent transactions may be far rarer than non-fraudulent transactions, resulting in a skewed distribution [65]. Unbalanced data can have a profoundly negative impact on the training of the global model, as it can bias the model toward the majority class and cause it to fail to recognize the minority class [14] accurately. Several methods can be employed to counter this issue, including data augmentation and class weighting. Yet, these approaches are not without issues in federated contexts, particularly where access to data by single clients is restricted [65].

**Figure 5:**
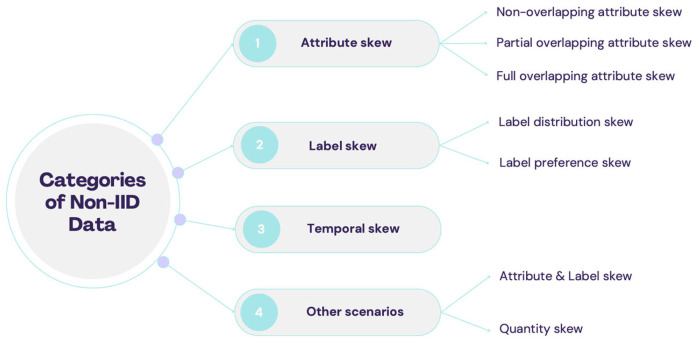
Categories of Non-IID Data [66].

A detailed explanation of the types of non-independent and non-identically distributed (non-IID) data in federated learning is given in [66] as shown in Figure 5, which shows how client data can differ in several ways:

1. **Attribute skew:** When clients have different feature spaces that may or may not overlap.
2. **Label skew:** When class labels or preferences differ between clients.
3. **Temporal skew:** When data changes over time.
4. **Attribute–label skew:** A combination of attribute and label skew.
5. **Quantity skew:** When clients hold unequal amounts of data.

### 2.2. Edge Computing Core Concepts

Edge computing is a distributed computing model that brings processing capabilities closer to data sources, e.g., sensors, mobile devices, and Internet of Things (IoT) endpoints. Contrasting with conventional cloud-based models, in which raw data is sent to central data centers, edge computing enables on-the-fly data processing to minimize latency and bandwidth usage, while also enhancing privacy [23]. Applications that require real-time responsiveness, such as autonomous vehicles, medical surveillance, and industrial automation, can be processed more effectively and with greater reliability by pushing intelligence to the network edge [25].

The fundamental ideas of edge computing are proximity, low latency, scalability, and reliability. Proximity enables processing data in the region where it was generated, minimizing delays and making it essential for time-sensitive applications [67]. Low latency enables applications such as self-driving vehicles [2] and augmented reality, where milliseconds matter for safety and usability [68]. Scalability is demonstrated through the decentralization of workloads from centralized clouds by distributing tasks across distributed nodes, balancing workloads, and enabling the enormous expansion of IoT devices. Reliability is facilitated through continuous service delivery, even in the presence of intermittent cloud [69].

Edge computing presents challenges despite its significant promise. The disparity in computational capacity and energy efficiency is caused by the heterogeneity of resources across devices [70]. Data breaches and adversarial attacks are security risks that are exacerbated by the decentralized organization of edge networks [71,72]. Also, orchestration, interoperability, and cost-effectiveness are among the significant issues to address when deploying large-scale systems [73].

In conclusion, edge computing marks the transition of cloud dominance to distributed intelligence. Its essence, comprising concepts of locality, latency reduction, scalability, and reliability, forms the basis of next-generation applications. In combination with federated learning, edge computing is a key enabler of privacy-protecting, real-time, and adaptable AI systems at scale.

#### 2.2.1. Cloud vs. Edge vs. Device Hierarchy

Edge computing provides a distributed computing model that processes data at a point closer to the source, minimizing latency and eliminating the need for high bandwidth commonly associated with cloud computing. Cloud computing is primarily centralized, with large volumes of data being submitted to the cloud for processing. The cloud offers vast computing resources, but it is prone to significant latency and bandwidth issues when working with time-sensitive applications [74]. Edge computing can address these problems by moving data processing to the network edge, where devices (e.g., IoT sensors, smartphones) can perform computations locally [23].

**Figure 6:**
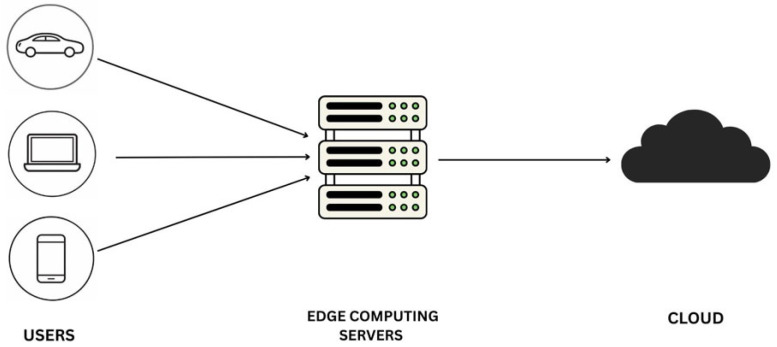
A Hierarchy of Cloud vs. Edge vs. Device.

The generally accepted edge computing hierarchy consists of three layers, as shown in Figure 6. The device layer comprises local devices, such as smartphones and Internet of Things (IoT) sensors, that generate data and may perform basic processing. The edge layer includes local servers or gateways that perform more complex calculations and make decisions [23]. Lastly, large-scale data storage, model training, and long-term analytics are not real-time computations and therefore use the resources offered by the cloud layer [19].

#### 2.2.2. Edge Computing Advantages

Minimizing latency is one of edge computing’s most significant advantages. Edge nodes reduce the time required to channel information in both directions between devices and remote cloud servers by processing data near the source [23]. This property is fundamental to real-time applications, including autonomous driving, industrial automation, augmented reality, and telemedicine, where a few-millisecond delay can compromise performance or pose safety risks [67]. For example, low-latency, rapid decision-making can be used to facilitate collision-avoidance systems and traffic optimization, especially in vehicles, through vehicular edge computing [75].

The other benefit is effective bandwidth utilization. Conventional cloud systems transmit raw data in bulk to centralized data centers, which can lead to network congestion and increased costs. Edge computing reduces the burden by filtering, aggregating, and preprocessing data locally and transmitting only the relevant or summarized data to the cloud [58]. This not only minimizes congestion in backbone networks but also ensures scalability as the number of billions of IoT devices continues to increase worldwide [25]. To illustrate, wearable sensors and devices can be used in healthcare monitoring to analyze patients’ vitals at the device level, sending only unusual patterns to the cloud for processing, thereby avoiding continuous data transmission [3].

Latency reduction, combined with bandwidth optimization, unlocks new possibilities in data-intensive fields through edge computing. The benefits of smart cities, intelligent transportation systems, and energy management infrastructure include shorter response times and greater efficiency in resource use [11]. In addition, edge computing enhances system resilience to low-throughput network connectivity by reducing reliance on limited or unreliable internet connectivity [70], ensuring reliable operation under unstable network conditions [76].

Moreover, the bandwidth performance of edge computing is an added advantage that reduces costs for both service providers and end users. Backhaul traffic can be reduced by network operators and organizations implementing edge solutions, thereby avoiding high cloud usage [67]. This makes edge computing particularly appealing for large-scale implementations where performance and cost-effectiveness must be balanced [77].

Another advantage of edge computing is that it enhances privacy and energy efficiency by keeping sensitive data locally and reducing unnecessary data transmission. It also reduces risks such as eavesdropping and breaches in cloud systems [78]. Lastly, edge computing enhances context awareness, enabling adaptation to real-world environments [79].

**Figure 7:**
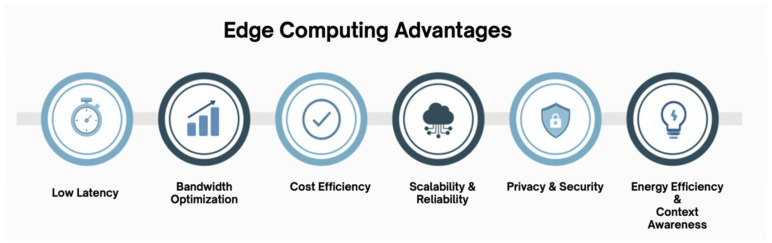
Edge Computing Advantages.

Figure 7 illustrates the key advantages of edge computing, including latency, bandwidth, cost efficiency, scalability, reliability, privacy, security, energy efficiency, and context awareness.

To conclude, the fundamental benefits of edge computing—reducing latency and optimizing bandwidth—are the technical basis for enabling real-time, scalable, low-cost applications. These strengths support the use of edge computing as a key infrastructure to support next-generation intelligent systems.

#### 2.2.3. Resource Constraints at the Edge

Resource limitations of edge devices are among the most critical issues in edge computing. The power, memory, and storage of these devices are typically limited. To illustrate, many IoT devices are designed to be cost-effective and energy-efficient, which limits their ability to run complex algorithms or store large amounts of data [67]. Additionally, many edge devices use batteries, making it difficult to perform continuous calculations without excessive power consumption. This necessitates the development of lightweight models [13] and resource-efficient algorithms that can execute the required tasks without violating the power and resource constraints of edge devices [12].

### 2.3. Synergy of FL and Edge Computing

#### 2.3.1. Federated Learning Is a Perfect Fit for Edge Environments

The ability to conduct distributed learning without violating data privacy makes Federated Learning (FL) highly suitable for Edge Computing (EC) settings [10]. Edge environments comprise devices such as smartphones, IoT sensors, and wearables, which are distributed across different locations and typically have limited computational capabilities. FL considers this by enabling devices to learn in place and transfer only model updates, not raw data, to a central server for aggregation. This is particularly important in edge environments, where it can be inefficient, expensive, or even impossible to send large volumes of data to a central server due to bandwidth constraints and privacy considerations [20].

Additionally, FL would be a good fit for edge environments because it does not require all data to be centrally located for collaborative learning [59]; this makes it easier to run data on the edge with lower latency [25]. This feature enables FL to be used to the fullest in systems that require real-time data processing, autonomous driving, real-time health monitoring, and smart home devices, where latency is a key factor [12]. The decentralization of FL also ensures that data remains on the device, which is critical for ensuring data sovereignty and compliance with privacy laws [71].

#### 2.3.2. System Architecture (Device-Edge-Cloud)

The Federated Learning architecture in edge computing is generally hierarchical, with three main layers: the **device layer**, the **edge layer**, and the **cloud layer**. The device layer consists of edge devices (smartphones, IoT devices, and other connected sensors) that produce and process localized data. Such devices can train local models with their data and transmit model updates (i.e., gradients) to the edge layer for aggregation [80].

The edge layer features local edge servers or gateways that receive model updates from multiple devices, combine them, and perform any necessary additional calculations [81]. Such edge devices may be closer to the source of data and provide real-time or near-real-time processing and decision-making, eliminating the need to transmit all data to the cloud [71].

More advanced applications of the cloud layer typically involve global model aggregation, long-term storage, and large-scale model training. Although edge computing can handle most tasks locally, the cloud serves as a central location where consolidated models across various edge devices are stored and further developed [82]. This cloud-edge-device architecture is both privacy-aware and high-performing, as it minimizes the volume of information passing through the network while ensuring real-time decisions are made at the edge [36].

#### 2.3.3. Applications (Smart Cities, Autonomous Vehicles, Healthcare IoT)

The ability to leverage decentralized devices and maintain data privacy is why federated learning is rapidly being deployed across many edge computing applications [81]. FL may be applied in smart cities to enhance traffic control, security monitoring, and environmental monitoring systems. Edge sensors (traffic cameras, street sensors, and weather stations) produce significant volumes of data, which can be trained at the edge, and FL can be used to jointly train models across them without necessarily centralizing the data [83].

FL allows vehicles to learn together using sensor and camera data from autonomous cars and prevents the transmission of sensitive information about vehicle positions and driver actions to central servers. Such a decentralized solution enables the vehicle to continually refine its driving model, adapt to novel circumstances, and share knowledge with other vehicles in real time [2], while preserving privacy and minimizing latency [39].

The case study presented in [2] uses FL to predict steering angles in real time for autonomous vehicles. The application demonstrates the feasibility of training high-performance models on edge hardware first, thereby reducing their dependence on centralized data collection. The authors emphasize mitigated latency and enhanced data privacy as primary advantages, particularly for deployments in areas with intermittent connectivity.

FL has found use in the healthcare IoT market for patient monitoring devices, where wearables and IoT devices continuously collect patients’ health data, including heart rate, blood pressure, and oxygen levels [69]. Local models can be trained to diagnose potential health risks, such as arrhythmia or heart attack, and the global model can be enhanced with information from multiple devices belonging to different patients [25]. This enables individualized medicine while ensuring that medical records containing sensitive information are never stored in the cloud [84].

Authors in [3] proposed a cloud-edge-based, personalized FL framework, FedHome, for in-home health monitoring in healthcare. They utilize an architecture for wearable devices to track patients’ health information through local training. The framework addresses the issues of non-IID data and device heterogeneity, which are significant concerns in the real-world deployment of FL. Likewise, Qayyum et al. [6] sed FL to perform COVID-19 diagnostics on any given multimodal medical data (e.g., CT scans, X-rays, and ultrasound), which are processed on medical edge devices. This method will help to identify it in time and provide data sovereignty.

FL has also been helpful in the utility sector. Taik and Cherkaoui [11] used edge-based FL in forecasting electrical loads in smart grid settings. Smart meters act as edge clients, predicting consumption patterns and sending model updates to a central server. In this analysis, superior prediction rates are achieved while meeting data protection standards.

**Figure 8:**
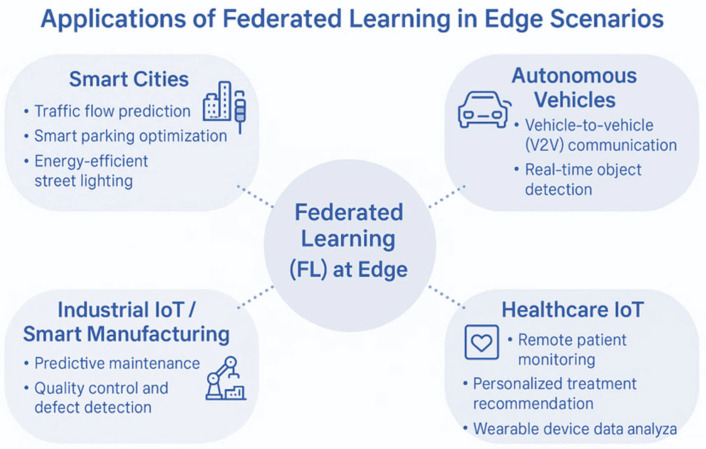
Application of Federated Learning in Edge Scenarios.

These applications, as shown in Figure 8, are widely used in various fields, including automotive, healthcare, and energy. Individual deployments will demonstrate how FL can be adapted to edge settings and highlight common limitations, including computational overhead and communication bottlenecks. Table 3 summarizes some applications of the Federated Learning system.

#### 2.3.4. Technical Challenges (Communication, Adversarial Attack, and Scalability)

Though Federated Learning (FL) has significant benefits in edge settings, the model also presents several technical challenges that should be met to be successfully deployed:

The most critical issues with Federated Learning systems are privacy, security, and scalability, particularly in edge environments where a large number of heterogeneous devices generate data [7]. Although FL guarantees that the raw data does not leave the machine, privacy leakage and model inversion attacks pose serious threats, as an attacker can still learn sensitive information from changes in the model [9]. Recent advances in secure aggregation and differential privacy provide approaches to ensure the safety of individual data contributions during model training [7]. However, such mechanisms are currently being optimized for edge contexts, where devices are resource-limited [84].

In addition, FL is vulnerable to adversarial attacks, including model poisoning and backdoor attacks, in which malicious clients can use local updates to model training to undermine the performance of the global model [12]. Federated adversarial training and homomorphic encryption are also under development, aiming to improve the security of the learning process and mitigate that a global model is not weakened by malicious actors [87].

Resilience to adversarial and faulty clients is another important issue in federated systems, particularly in edge computing settings [12]. Given that edge devices can be deployed in uncontrolled environments, their operation is susceptible to malfunctions caused by hardware or software bugs or attacks [71]. Adversarial clients may maliciously manipulate the global model via poisoning local updates or backdoor attacks [9], in which the adversary injects triggers that activate only when a particular condition is met [88].

There is always a scalability problem in federated learning systems as the number of edge devices grows. Federated learning models should be able to handle multiple clients with locally available datasets. The heterogeneity of the devices and data among these clients may also complicate scaling. Hierarchical aggregation and adaptive client sampling are two strategies suggested to scale up, though they will need to be optimized for edge environments, where resources are scarce [39].

Energy-aware, resource-efficient federated learning is a promising future direction, given the resource constraints of edge devices, particularly those powered by batteries [89]. The computational power and memory required to train large models or process large datasets are often expensive and may not be readily available in sufficient quantities on edge devices. To address this, model compression, sparsification, and parameter sharing are being implemented to reduce training computational cost [12].

Additionally, adaptive client sampling can enable the system to serve more resource-intensive clients with larger computational resources or more valuable information, *thereby improving training efficiency* [12]. Such mechanisms are crucial for making FL systems viable in resource-limited settings, such as smart homes, autonomous vehicles, and medical IoT, where energy efficiency and resource utilization are paramount [71].

Bringing cloud, edge, and federated learning (FL) together into a hybrid solution provides an opportunity to overcome the resource constraints and scalability challenges of federated systems [90]. In this hybrid model, edge devices can perform local training and initial processing, while edge servers bundle model updates and perform more complex operations. The cloud layer, on the other hand, provides centralized aggregation and large-scale model training. Such a hierarchical design will maximize resource utilization, balance computational load across devices [37], edge servers, and the cloud, and address latency and communication overhead [71].

Multi-tier aggregation is also possible with hybrid systems, in which clients’ updates are first aggregated at the edge layer, and then a subset is sent to the cloud. This method has been shown to improve the scalability and efficiency of FL systems, particularly in applications such as IoT, smart cities, and autonomous vehicles [84].

As Federated Learning (FL) continues to emerge, standardization is required for methods, evaluation metrics, and frameworks. However, no consensus exists on the mechanisms for evaluating the performance and security of federated models. Thus, it is challenging to compare solutions across various domains and application scenarios. There is also a lack of open research directions that address data and client heterogeneity, as well as privacy-preserving mechanisms, in federated systems within edge computing environments.

The next step in future studies is to develop standard benchmark datasets, evaluation systems, and protocols that enable the comparison and deployment of federated systems across various industries. Increasingly, there is a need to research open-source federated learning platforms to support more collaborative work and ensure interoperability across different devices and infrastructures.

Federated Learning (FL) has attracted significant research attention due to its practical applications in edge computing, particularly because it enables real-time intelligence without requiring data sharing. Several case studies demonstrate the application of FL to support on-device learning in dynamic, resource-constrained environments, providing insights into its capabilities and limitations.

## 3. Vulnerabilities and Threat Model in FL -Edge Systems

Although Federated Learning (FL) can be highly beneficial for privacy protection because unencrypted data does not leave the machine, a distributed, decentralized FL architecture raises several new security and privacy concerns. Not only do these vulnerabilities affect the integrity of models, but also the confidentiality of local datasets, which is of utmost importance in edge computing, where devices tend to be resource-limited and less secure.

Model poisoning represents a significant threat vector in FL, in which malicious clients send poisoned gradients to manipulate the behavior of the global model [8]. As an example of such an attack, Bagdasaryan et al. [9] illustrate how to conduct backdoor attacks in FL by adding poisoned updates that alter model responses when applied to specific inputs. Such attacks are discrete and pose significant challenges for the robustness of federated learning systems.

The other major weakness is data leakage via gradients. Since gradient information is used in FL updates, adversaries can infer sensitive input data from gradient data [10]. The attack proposed by Zhu et al. [10] demonstrates that a gradient can be exploited through an iterative optimization process to reconstruct gradient descent and leak visual or textual content from private datasets. In the same context, Melis et al. [91] point out an unintended feature-leakage issue, where common updates used to update a shared model incidentally encode similar-level patterns in the training data.

FL robustness is also threatened by Byzantine attacks, in which clients act randomly or maliciously. Bhagoji et al. [92] experimentally demonstrate the impact on performance of such adversaries, and Lyu et al. [7] argue that Byzantine behaviors are a key threat model in federated learning. Such threats increase in edge environments when there is a higher probability of either physical device compromise or network spoofing.

Additionally, there are also inference attacks to be concerned about. The adversaries can also target the extraction of features from nearby collections or the inclusion of samples through multiple model queries [12]. This is especially serious in care or monitoring installations installed on the edge.

### 3.1. Vulnerability Sources

Although edge computing enables low-latency services and efficient bandwidth utilization [13], it introduces several sources of vulnerability that should be systematically understood to ensure deployment security and resilience [12]. One of the first and most basic sources is decentralized control, as edge networks distribute computational intelligence across heterogeneous nodes with partial autonomy, thereby minimizing the efficacy of centralized governance and homogeneous security protection. This decentralization tends to introduce inconsistent authentication, patching, and configuration policies, leaving gaps that attackers can exploit to escalate privileges or disrupt operations [67]. A second weakness arises from untrusted clients, as edge computing is directly in contact with a broad range of end devices, including IoT sensors, smartphones, and embedded systems, many of which cannot be deemed trustworthy due to weak security postures or exposure to harsh environments [81]. Infected or malicious clients can provide tainted information, mount inference attacks, or affect joint learning, and federated and collaborative environments are especially vulnerable, as a single malicious actor might jeopardize the integrity of the global task [36].

Communication channels are the third most vital area, as they are the primary means of data transmission between end devices, edge nodes, and cloud platforms [76]. These channels, typically wireless and resource-constrained [93], are susceptible to eavesdropping, replay, and man-in-the-middle attacks [94], especially when high overhead prevents the use of robust cryptographic protocols [95]. Lastly, there is another systemic weakness: limited device resources, with edge devices often having limited computational capabilities, memory, storage, and energy budgets [84], which limits the feasibility of computation-intensive security mechanisms, particularly blockchain-based verification and privacy-preserving techniques that rely on intensive cryptographic operations [95].

These constraints subject devices to denial-of-service, resource denial-of-service [94], and side-channel attacks [96] that adversaries can use to impair service quality or cause system failures.

**Figure 9:**
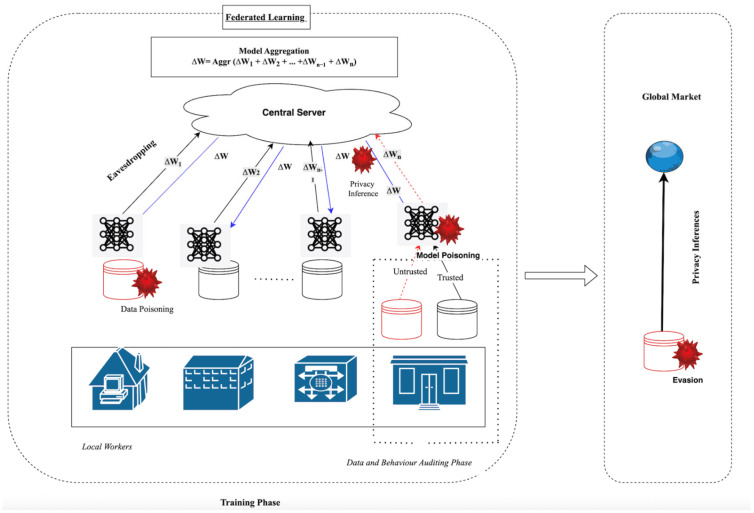
*Adversarial* threats in federated learning at the Edge [97].

To conclude, the combination of the four categories constitutes the threat environment of edge computing: decentralized control, lack of trust among clients, untrustworthy communication channels, and limited resources. Such vulnerability sources need to be mitigated with lightweight, adaptive, distributed defense mechanisms that are sensitive to the dynamism and heterogeneity of the edge environment so that the performance benefits of edge intelligence are not compromised by systemic insecurity [74]. Figure 9 illustrates the primary attack surfaces that emerge during the training and prediction phases of FL systems, including data poisoning, model poisoning, privacy inference, eavesdropping, and evasion [97].

### 3.2. High-Level Threat Model in Federated Learning

The scope and severity of the potential attack can be defined by analyzing the adversary’s goals, capabilities, and knowledge, which, in turn, can characterize the high-level threat model of edge computing environments [97]. The objectives of the adversary can include breaching data security, compromising model integrity, obtaining unauthorized access to sensitive information [7], or interfering with service availability [98]. In most instances, attackers compromise privacy by deriving raw data from gradients [10] or leaked metadata, or by introducing malicious updates that reduce the performance of global models in collaborative learning [9].

Alternative ends include monetary rewards through denial-of-service attacks [99], edge-based transaction misuse in IoT-enabled edge services [100], or even using insecure nodes as participants in cyberattacks [101]. The capabilities of the adversary can range from local eavesdropping on communication channels, which is the lowest level [72], to complete damage to edge devices or servers. For example, a strong adversary can perform poisoning or backdoor attacks by manipulating multiple clients [8]. In contrast, less advanced attackers can use resource exhaustion to mount flooding or jamming attacks [72]. Attackers in edge-cloud systems can use compromised infrastructure to manipulate model updates and influence the aggregation of the global model [102].

Lastly, the effectiveness of attacks depends on the adversary’s knowledge. A white-box adversary possesses specific knowledge of system architectures, parameters, and defenses, enabling them to launch targeted attacks, such as gradient inversion stealthily [10] or model extraction [57]. Conversely, gray-box adversaries have access to partial information, i.e., they may know the protocol used by the learning process but not the local data distributions [91]. In contrast, black-box adversaries have access to observable input-output behavior [103]. The environments along the edges are especially vulnerable, as the absence of centralized control and the diversity of devices increase the likelihood that attackers can develop partial knowledge via side channels, insecure firmware mechanisms, or weaker-secured APIs [104].

A combination of these aspects—goals, capabilities, and knowledge—demonstrates that edge computing and federated learning ecosystem adversaries can be opportunistic attackers who exploit vulnerable devices or well-funded adversaries who organize coordinated, multi-vector campaigns. Creating efficient defenses thus involves predicting a variety of adversary personas and matching shielding solutions to the system’s resource constraints, communication restrictions, and privacy-saving requirements [19].

**Figure 10:**
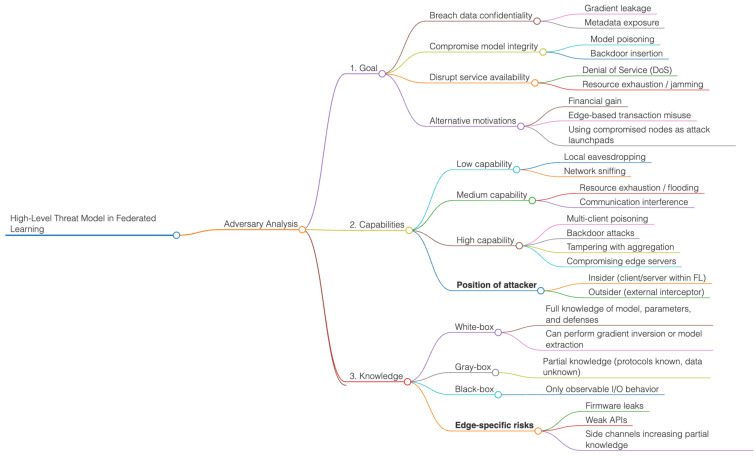
Summary of *the high-level* threat model in federated learning at the Edge adversaries. Edge-specific risks such as firmware leaks and weak APIs further amplify these threats.

The diagram in Figure 10 illustrates the hierarchical structure of adversary analysis in FL-edge environments. It classifies threats into three main dimensions: *goals*, which include violations of confidentiality, integrity, and availability; *capabilities*, ranging from low-level eavesdropping to large-scale poisoning and tampering with aggregation; and *knowledge levels*, including white-box, gray-box, and black-box.

## 4. Taxonomy of Adversarial Attacks on Federated Learning

To understand adversarial attacks on federated learning systems in a cloud computing environment, we categorized attacks based on their intended impact on the system. Based on the threat model discussed earlier, we divided the attacks into four main attacks:

- Integrity attacks: types of attacks that aim to manipulate the training process or corrupt the global model behavior. As a result, they undermine the model’s validity.
- Privacy attacks: types of attacks that target sensitive customer data or local data derived from shared updates or model outputs.
- Availability attacks: types of attacks that disrupt or degrade the learning process, system reliability, or client participation.
- Communication-based attacks: types of attacks that exploit vulnerabilities in transmission channels, coordination mechanisms, or control messages.

Because of the interconnected nature of federated learning workflows, which include local training, update transmission, aggregation, and model dissemination, many attacks do not clearly fall into just one category. Communication tampering or replay attacks, for example, primarily interfere with how model updates are exchanged, but the damage they cause often manifests as degraded model integrity or reduced system availability. Similarly, poisoning attacks intended to corrupt model behavior can also lead to privacy leakage over time. For clarity, the attacks were categorized based on their primary and immediate impact on the system.

**Figure 11:**
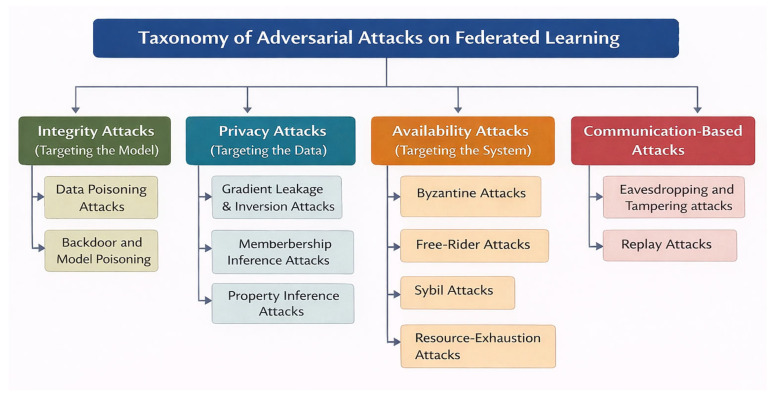
Illustrates the taxonomy of adversarial attacks on federated learning systems.

This classification enables the analysis of adversarial behavior and the design of appropriate defense mechanisms. Figure 11 illustrates the taxonomy of adversarial attacks on federated learning systems.

### 4.1. Integrity Attacks (Targeting the Model)

#### 4.1.1. Data Poisoning Attacks

One of the most prevalent attacks in federated learning and edge computing systems is data poisoning, where attackers intentionally modify training data or model updates to compromise the integrity and reliability of the global model [8]. In these attacks, malicious clients either inject poisoned samples into their local datasets or generate adversarial gradients that cause the aggregation process to favor them, thereby worsening accuracy or causing desired misclassifications [9].

The general types of poisoning attacks include label-flipping attacks, in which malicious users deliberately mislabel the training data to distort the model’s decision boundaries. Indicatively, Bagdasaryan et al. [9] showed that backdoor poisoning in federated learning can be stealthy and undetected by aligning malicious updates of benign directions of model updates. These risks are even higher in federated systems, where there is no centralization and the privacy of the information shared by clients is ensured, since the updated model is not directly visible in the client dataset and may be difficult to identify malicious contributions [12].

Additionally, the non-uniformity of data distribution across clients makes it more challenging to detect poisoned updates, as natural non-IID data can be similar to adversarial manipulations [105]. Data poisoning may also be an initial step towards larger attacks, such as membership inference or model recovery through undermining global robustness. Byzantine-resilient aggregation schemes to downweight outliers, anomaly detection schemes to sieve out suspicious updates, and clustering-based recalibration schemes to isolate malicious clients are some of the defensive mechanisms [15,47].

Also, some vectors of poisoning can be mitigated using methods such as differential privacy [106] or verifiable secure aggregation [84], albeit at the expense of accuracy or efficiency. These developments notwithstanding, the challenge of poisoning remains difficult to overcome due to the adaptive mechanisms adversaries employ, including increasing attack strength to prevent detection or coordinating across multiple vulnerable clients [107].

To conclude, data poisoning attacks exploit the collaborative nature of federated and edge learning systems, posing threats to model accuracy, trustworthiness, and safety. The solution to them requires comprehensive defense measures that incorporate secure aggregation, anomaly detection, and privacy-preserving mechanisms tailored to resource-constrained, decentralized settings [19].

#### 4.1.2. Backdoor and Model Poisoning Attack

Backdoor injection and model poisoning are among the most dangerous attack vectors in FL, as attackers can design malicious updates that cause the global model to misbehave. Unlike overt attacks that undermine model performance, backdoor attacks are subtle and feature a trigger mechanism that activates them for specific inputs. Bagdasaryan et al. [9] showed that a client adversary could poison a local FL training client, leading the final global model to misclassify a data sample containing a trigger pattern (a patch of pixels in the image). The attack was highly accurate on clean data, making it unnoticeable in standard validation. Poisoning can be performed via data manipulation (e.g., label flips [108]) or gradient manipulation (e.g., scale updates), both of which affect the ultimate model parameters during aggregation. Such threats are particularly severe in critical systems, such as self-driving vehicles that may encounter a mislabeled stop sign or healthcare diagnostics that may produce false forecasts of specific symptoms.

Such attacks, according to Lyu et al. [7], can be divided into untargeted and targeted poisoning, where the objective is to induce general degradation or misbehavior at a particular point. As recently demonstrated by Manzoor et al. [109] and Chelli et a. [110], even simple aggregation-based defenses, such as FedAvg, are vulnerable to backdoor attacks because they are susceptible to outlier updates. Furthermore, in environments where FL is deployed across heterogeneous systems (non-IID data, diverse client capabilities), it is more difficult to distinguish between poisoned and benign updates. This creates a need to develop strong and trust-sensitive aggregation schemes as a priority in a secure FL implementation. Some techniques that directly address this vulnerability and are effective include complementary defense frameworks such as FLIP [48] and trust-aware aggregation schemes such as FLTrust [13].

### 4.2. Privacy Attacks (Targeting the Data)

#### 4.2.1. Gradient Leakage and Inversion Attacks

Inversion and gradient-leakage attacks pose serious threats to the privacy of federated learning and edge computing systems, which exploit the communication of gradients or model updates to reconstruct sensitive client data. In contrast to data poisoning, which aims to compromise training procedures, gradient leakage attacks seek to compromise confidentiality by revealing raw training samples or sensitive features of the gradient information the aggregator was supplied with, despite it appearing harmless. Zhu et al. [10] demonstrated that a single gradient update can enable the recovery of detailed data from images formed during the private training procedure. This approach, known as Deep Leakage from Gradients (DLGs), demonstrates that under such conditions (a known model architecture and access to gradients), a malicious party can obtain highly accurate reconstructions of inputs.

Similarly, Melis et al. [91] demonstrated that collaborative learning may lead to unintended feature leakage, exposing sensitive demographic features such as gender or location. A survey study [13] suggests that interpreters (such as FL participants) may draw inferences from latent data representations, particularly in arrangements involving vision and language models. Such attacks are more robust in federated systems, where attackers can be either the central server (honest-but-curious) or the target client and can leverage partial gradients to run gradient inversion, optimizing synthetic inputs that produce gradients similar to those of the target data [111]. Leakage of gradients is a serious concern in healthcare settings, where patient scans or clinical records may be compromised, and in the financial sector, where sensitive user transactions may be at risk.

Inversion relies heavily on the adversary’s knowledge of the underlying model architecture and training procedure such that adversaries with a complete understanding, i.e., white-box adversaries, can obtain near-perfect reconstructions. In contrast, adversaries with only black-box knowledge can still extract rough information through iterative approximation [57].

This issue is exacerbated in non-IID federated environments, where clients are skewed, allowing adversaries to distinguish client-specific attributes [46,112]. The suggested countermeasures include the use of differential privacy protocols that add controlled noise to gradients [106], secure aggregation protocols that obscure individual entries [84], and gradient-compression protocols that minimize the fidelity of transmitted updates [45]. Privacy, accuracy, and computational efficiency, however, are frequently subject to trade-offs by these defenses, especially when resources are limited by edge devices [15]. Unfortunately, side information can still be used by adaptive adversaries to overcome naive defenses or to exploit tone inversion to evade them.

To conclude, the gradient leakage and inversion attacks indicate the inherent conflict between federated learning and privacy maintenance through collaborative training. These attacks underscore the timeliness of such a solution by demonstrating that even gradients can serve as proxies for the underlying data, inadvertently disclosing sensitive information in a distributed learning system [12,19]. Therefore, it is crucial to protect gradients using differential privacy, encryption, or robust aggregation to ensure that FL can effectively achieve its privacy-preserving goals.

#### 4.2.2. Membership Inference Attacks

Membership inference attacks (MIAs) pose a significant privacy risk in federated learning and edge computing systems: they aim to determine whether a particular sample of data was included in a client’s training set. In contrast to poisoning or gradient inversion attacks, which aim to manipulate or reconstruct data, MIAs exploit statistical variations in model responses or updates to obtain membership data and, as such, violate personal privacy [33].

In a standard MIA, the adversary also exploits the fact that, in many models, performance on training samples is usually bimodal compared to that on unseen data, scoring higher on memorized inputs or lower on unseen data [33]. Using these differences in shared gradients, parameters, or prediction confidence, the attacker can confidently predict with nontrivial accuracy that a target record was used during training [113]. MIAs may be initiated by central servers, which are honest-but-curious adversaries with access to client updates, or by malicious clients that observe aggregated models round by round to determine whether a given sample is being used [57,112].

This risk is worsening in federated and edge contexts, where non-IID data distributions and small client datasets amplify model overfitting [46], which becomes more pronounced. For example, adversaries can leverage federated healthcare models to determine whether sensitive patient medical records can be used, raising serious ethical and legal concerns [114]. Researchers have suggested using defenses such as differential privacy, which introduces noise to gradients to decrease membership distinguishability [106]; secure aggregation, which does not expose individual updates; and regularization-based techniques, which mitigate model overfitting [115]; partial sharing mechanisms and anomaly detection techniques [113]

Despite these techniques, they entail trade-offs among accuracy, communication cost, and computational overhead, which can be very demanding [15]. In addition, adaptive MIAs may also utilize auxiliary information, such as shadow models, data distributions, or partial gradient statistics, to circumvent naive defenses [113]. To conclude, MIAs highlight the privacy-utility trade-off in federated learning and edge computing: although the paradigm ensures the locality of raw data, the iterative sharing of model updates unintentionally results in the dissemination of statistical data that adversaries can exploit [19]. To address this, it is essential to incorporate powerful privacy-preserving techniques into the federated learning cycle to protect client privacy and ensure regulatory compliance for sensitive tasks.

#### 4.2.3. Property Inference Attacks

Property inference attacks (PIAs) are a confidential yet influential privacy threat in federated learning and edge computing systems, where adversaries aim to infer global or customer-specific properties from training data that are not directly relevant to the learning task. PIAs are used to infer latent variables, such as demographics, behaviors, or other sensitive patterns, in datasets. This differs from membership inference, which aims to infer the existence of individual samples, and gradient inversion, which aims to recover raw data [39].

For example, Melis et al. [91] demonstrated that, even with a completely different classification task, adversaries could infer unintended properties, such as gender, ethnicity, or specific identities, in an image dataset. Likewise, accidental leakage of features may expose the relationship between assistive features and gradient distributions, revealing sensitive information about a patient such as Yelp healthcare, or a user’s whereabouts in mobile services [112], Honest-but-curious servers can also launch PIA, meaning they observe changes in clients during training rounds, or malicious clients can analyze aggregated models to tell property-related changes in signals [116]. It is particularly severe in federated and edge settings when using non-IID data, because skewed distributions may exaggerate property-specific gradients [64] that adversaries can exploit to make inferences. In addition, PIAs can be applied not only to direct attributes but also to behavioral or temporal properties, e.g., to determine whether users perform specific routines based on device usage data [39].

Privacy-preserving methods like differential privacy (adding gradients with carefully controlled amounts of noise to obscure property-related information) [106], secure multi-party aggregation (hiding the individual client contributions) [84], and any form of adversarial regularization (expressly discouraging the encoding of unwanted properties in a model) [12] can be used to mitigate PIAs.

However, these methods are often concerned with trade-offs among robustness, model utility, and computational efficiency, particularly when operating with the limited resources of edge devices [15]. In reality, adaptive adversaries can still evade defenses by utilizing side information or auxiliary datasets to uncover correlations in property patterns, and this risk remains uneliminated [117]. Overall, property inference attacks demonstrate the susceptibility of federated learning systems to privacy breaches that extend beyond explicit training objectives, underscoring the need to design privacy-enhancing mechanisms that mitigate both direct and indirect information leaks in decentralized settings.

### 4.3. Availability Attacks (Targeting the System)

#### 4.3.1. Byzantine Attacks

One of the most documented and structurally threatening attacks of FL is the Byzantine attacks. Such attacks include clients that are programmed to act arbitrarily or maliciously and to undertake unpredictable, potentially corrupted updates that damage the learning process. Byzantine nodes can perform as well as send random gradients or symbolically revised gradients, up or down, or, in some situations, send upgrades that carry an adversarial goal [14]. Bhagoji et al. [92] noted that a small proportion of Byzantine clients would make training unstable and lead to convergence failure [14]. This risk is particularly pronounced in asynchronous training systems [118] and cross-device FL deployments [55] where participation is uneven and authentication is loose.

Here, the server is not always able to distinguish in-line-of-duty variations in updates (caused by non-IID data) from adversarial noise. Traditional techniques, such as FedAvg, are attractive targets for such attacks because they treat all client updates equally. To alleviate these threats, robust aggregation functions such as Krum [9] and Multi-Krum algorithms, as well as the trimmed mean, have been proposed by filtering or weighting updates using a distance function over statistics. However, as Dong et al. [13] observe, such defenses typically assume a malicious client rate and do not generalize well to the heterogeneity of real-world data [51]. Mitigating Byzantine risks is not only a question of correctness in industry or innovative infrastructure applications where FL is deployed in safety-critical operations, but also a question of integrity in operations.

#### 4.3.2. Free-Rider Attacks

The problem of free-rider attacks is a particular threat in federated learning and edge computing, as malicious agents can exploit the collaborative training structure to obtain the rewards of the global model without providing valuable local updates [118]. Compared to integrity and privacy threats such as poisoning or inference attacks, free-riding compromises the systems of equality, efficiency, and credibility in federated learning ecosystems [119]. In a standard free-rider game, a competitor can provide either random updates, outdated parameters, or no updates and still receive the aggregated global model in every training round [112]. This behavior enables the attacker to save on computational, storage, and energy resources, while honest clients expend significant effort on local training [120].

Additionally, there are strategic and opportunistic attacks, in which adversaries can exploit the statistical distribution of benign updates by pretending to follow it or by deliberately withholding contributions, respectively [23]. By disrupting the overall quality of the global model in this way, such attacks reduce the adequate amount of training data used, which is especially detrimental in non-IID environments where diversity of contributions is essential to model generalization [121]. Federated learning is a decentralized system that further worsens the problem, as it is not always possible to verify the authenticity or usefulness of each client’s contribution without violating privacy guarantees [35].

Several countermeasures have been proposed to reduce free-riding, including reputation-based systems that assign trust marks to clients based on their past reliability [120], secure contribution auditing that measures the similarity of updates to expected distributions, and incentive systems that reward valid participation and penalize non-contributor [30]. Cryptographic protocols, such as zero-knowledge proofs, have also been proposed to enable clients to demonstrate that calculations are performed locally without exposing raw data [84]. Detection mechanisms, such as FRAD, are extend secure contribution auditing by identifying free-rider behavior [122].

Nonetheless, these mechanisms add overhead to computation and communication, making them challenging to implement in edge environments with limited resources.

To conclude, free-rider attacks exploit the collaborative nature of federated learning, allowing attackers to generate benefits for the system without incurring the corresponding costs, thereby undermining the system’s efficiency and trust model. To overcome this challenge, lightweight yet verifiable contribution-validation frameworks should adopt a balanced approach to accountability, privacy protection, and system scalability.

#### 4.3.3. Sybil Attacks

Sybil attacks exploit the open, decentralized architecture of federated learning, where uncontrolled clients lack a central authority. A Sybil attack happens when an opponent designs a large number of false clients (Sybil nodes) that may take part in the training process (enlarging their effect on the global model). Such malicious clients can gather to introduce poisoned updates, impose biased learning goals, or sabotage aggregation by establishing a majority of votes [123]. Lyu et al. [7] and later supported by [38] listed Sybil attacks as a special type of poisoning, which multiplied the weight of the adversary in the aggregation phase. Because FL is often designed so that client subsets are randomly selected in each round, especially in large-scale settings or across devices, it becomes challenging to identify when two participants are under the control of a single player. This manipulation is even more effective when combined with backdoor or Byzantine behaviors, enabling attackers to control the direction and frequency of the modified updates [124]. FoolsGold [125] is a reputation-based defense mechanism that identifies clients whose updates exhibit excessive gradient similarity and reduces their influence during aggregation.

#### 4.3.4. Resource-Exhaustion Attacks

Resource-exhaustion attacks, also known as denial-of-service (DoS) or distributed denial-of-service (DDoS) attacks, pose a significant threat to federated learning and edge computing systems by overwhelming systems and computers with excessive computational or network requests. Resource-exhaustion attacks, rather than compromising integrity or privacy, unlike poisoning or interference attacks. Instead, they are designed to undermine availability and reliability so that honest clients and servers can no longer effectively engage in training or inference processes, as is the case with poisoning or inference attacks [126].

Federated learning can also be exploited to synchronize attacks by flooding the aggregator with invalid or significant updates, resulting in computation or storage bottlenecks [34]. Similarly, attackers can exploit resource exhaustion—limited CPU cycles, memory, bandwidth, and battery capacity—at edge nodes to overload the edge computing system, resulting in dropped connections, slow responsiveness, or even device crashes [127].

This attack is especially harmful to applications where latency matters, such as autonomous driving, medical monitoring, and the automotive industry, where downtime directly affects safety and performance risk [2]. Adversarial goals can also be employed in conjunction with resource exhaustion: for example, attackers can disrupt defense systems by overloading resources and simultaneously perform data poisoning or inference attacks [8]. Additionally, edge systems are decentralized and heterogeneous, increasing their vulnerability because weaker nodes can be targeted during selection, leading to cascading failures and disrupting the larger network.

Some of the suggested countermeasures include lightweight intrusion detection systems tailored for devices with limited resources [34]; traffic shaping and throttling to filter abnormal network loads [102]; and adaptive client selection in federated learning that prioritizes trustworthy participants and excludes suspicious ones [7]. Nevertheless, these defenses are usually traded off against scalability and energy efficiency, especially when used in large-scale IoT systems [71].

To conclude, resource-exhaustion attacks are inherent to the nature of edge and federated infrastructures, which are inherently limited in terms of computational, communication, and energy resources, thereby compromising service availability and stability. Their handling needs complicated, adaptive, and low-overhead defense systems capable of balancing resilience with the strict latency and efficiency demands of the next generation of distributed AI systems.

### 4.4. Communication-Based Attacks

#### 4.4.1. Eavesdropping and Tampering Attacks

Eavesdropping and tampering are among the most fundamental communication-level threats to federated learning and edge computing systems, which exploit weaknesses in the exchange of updates, parameters, or control signals between clients, edge servers, and central aggregators. Eavesdropping is a passive method of enemy interception of communications, in which an enemy intercepts channels to obtain unauthorized access to confidential information, such as gradients, model parameters, or metadata [128].

Although raw client information is retained in federated learning, gradients may contain extremely sensitive information about local datasets that adversaries can use to reconstruct inputs, infer membership, or infer hidden properties [91]. This risk is further compounded by wireless and heterogeneous topologies and edge communication infrastructure, since the adversary can exploit insecure Wi-Fi, cellular, or vehicular networks to initiate massive-scale surveillance. Tampering, on the other hand, is a form of active interference of communication, in which attackers inject, modify, or retransmit updates sent through communication channels to corrupt the global learning process or perturb edge services. Tampered gradients in federated learning can be used both as a means of data poisoning or backdoor attack and as a way of weakening model integrity or inducing malicious behaviors.

In edge systems, attackers may target configuration changes, control requests, or firmware updates, which can cause a device to malfunction or trigger a cascading failure in critical infrastructure. These two attacks are particularly hazardous in healthcare and autonomous driving, which are highly sensitive contexts where the loss of confidentiality and integrity directly translates into safety risks [2]. The suggested methods to resist eavesdropping include secure aggregation protocols that obscure personal updates before aggregation [84] and differentiating privacy methods that attenuate the sensitivity of any shared gradients [39]. Cryptographic integrity checks, such as digital signatures, hash-based message authentication codes, and blockchain-based logging, have been proposed as countermeasures against tampering to ensure that updates are not altered in transit [129].

Also, anomaly detection systems can track the severity of abnormal deviations that may signal tampering in [94]. However, such defenses tend to incur computational and communication overhead, posing a challenge for resource-constrained edge devices [15]. To conclude, eavesdropping and tampering pose threats to confidentiality and integrity, respectively, by intercepting communications through both silent and active means. The two emphasize the need to develop strong yet lightweight, verifiable communication security standards that maintain privacy and trust in distributed intelligence systems.

#### 4.4.2. Replay Attacks

Replay attacks, in contrast, take the form of reposting updates from earlier rounds to cheat the server or delay learning. Although they are not as damaging as Sybil attacks in a specific setup, replay attacks may distort the model’s convergence path, amplify outdated knowledge, or conceal the existence of other malicious activities. The attacks are especially applicable to non-synchronous FL systems, where not all clients can communicate simultaneously, and previous updates can be buffered or queued when connection reliability is uncertain [7]. Martinez Beltran et al. [130] noted that FL systems are vulnerable to timing- and topology-based attacks, especially in mobile or IoT environments. Recent protection efforts against replay attacks include client authentication standards, trust ranking, anomaly detection, and moving-target defense [131]. Nevertheless, such techniques introduce complexity and overhead in terms of the number of computations, which, in turn, become unfeasible to implement due to limitations in lightweight edge deployments. Thus, mechanisms against replay attacks represent a trade-off between model security and system scalability.

## 5. Taxonomy of Defense Mechanisms

### 5.1. Robust Aggregation Strategies

One of the first and best-studied defense mechanisms in federated learning is robust aggregation methods. They are primarily designed to mitigate the impact of corrupt or noisy client updates during model aggregation. In conventional FL environments, the server uses the simplest form of computation, such as averaging all updates (e.g., FedAvg [67]). The problem, however, is that this method presupposes that all clients are trustworthy and cannot lie; therefore, it may be prone to poisoning and Byzantine behavior. To counter this, Blanchard et al. [14] proposed the Krum algorithm, which selects the client update closest to the majority with respect to the Euclidean distance, thereby downplaying the effect of outliers. Krum is not very efficient in settings with few attackers; it is computationally intensive and sensitive to client heterogeneity.

Other algorithms that followed it to enhance scalability and robustness against larger attack ratios are Multi-Krum, Trimmed Mean, and Median Aggregation. These techniques combine a subsample of updates (by neighboring) or involve statistical smoothing, which discards outliers. Dong et al. [13] tested these approaches against different attack scenarios. They concluded that, although they perform better than FedAvg in terms of resiliency, their performance will degrade in non-IID scenarios, where honest client updates will tend to diverge from the global update distribution [97]. Moreover, the strong aggregation technique may require prior assumptions about the number or proportion of malevolent clients, which is not true in real-world conditions.

In federated learning, defense mechanisms based on trust leverage behavior baselines or trust scores to estimate and regulate the credibility of participating clients during aggregation. These mechanisms extend beyond statistical blocking to qualitative judgments about client updates, and in many cases, may utilize prior information, out-of-band metrics, or past predictability. The main principle is to detect destructive or unusual behavior and shut it down by assigning different weights to client contributions depending on their reliability. This is especially applicable in settings where client involvement is dynamic and potentially antagonistic, such as in cross-device or open FL settings.

Among the most popular frameworks used in this aspect, one can note the FLTrust framework proposed by Cao et al. [132]. It is a strategy that presupposes that a server has access to a small, trusted dataset and uses it to create a clean reference update. The baseline is compared to all client updates with cosine similarity. Significantly deviating updates are down-weighted or not aggregated. FLTrust outperformed traditional robust aggregation methods in terms of robustness against Byzantine attacks, including targeted poisoning and backdoor attacks. Its effectiveness stems from introducing a server-side root of trust to guide the aggregation process. But the use of FLTrust depends on the existence and applicability of the trusted dataset, which is not always viable in more decentralized systems.

Another approach, called FLIP (Federated LearnIng Provable defense framework), proposed by Zhang et al. [48], involves robustly training a model via FLIP by adversarially hardening client models during local training via trigger inversion and adversarial training. The framework does not rely on updated similarity or client reputation to calculate the aggregation weights. It mitigates the impact of compromised clients via weakening backdoor features in the global model, particularly when there are persistent attack scenarios.

Along with these algorithmic methods, some frameworks operate reputational systems, where clients earn trust scores that subsequently inform operations based on their historical performance or behavior. Such a score determines their pick in subsequent rounds or the weight of their contributions. Trust-based mechanisms, although promising, may raise concerns regarding scalability, fairness, and the decentralization of trust bootstrapping. In heterogeneous FL settings, honest clients whose data are atypical are also likely to be falsely labeled as untrustworthy, potentially leading to client exclusion and degraded generalization. Together with statistical aggregation and anomaly selection, they enable an adaptive, context-aware learning process that is better able to resist advanced adversary schemes. The design, however, should provide a strict balance between trust estimation accuracy and the demand of inclusiveness and computational feasibility in edge settings [131].

### 5.2. Privacy-Preserving and Gradient Clustering Techniques

Privacy-sensitive approaches to federated learning (FL) are designed to ensure that sensitive data is not inferred from model updates, while ensuring that the server (or peers) has a useful global model [21]. The most severe threat is gradient leakage, where an attacker uses common gradients to reconstruct training cases, infer membership, or identify confidential traits [10]. The most common practical defenses can be divided into three disjoint types: (i) update sanitization and outlier suppression; (ii) formal privacy mechanisms, and (iii) cryptographic protection of the aggregation channel. Gradient clustering and update sanitization: these techniques operate under the premise that harmful gradients are statistically (or structurally) distinct from benign gradients in some manner, typically in their distribution, direction, or uniformity across training steps. By aggregating similar gradients and applying sanitization methods, the system can silence outliers and remain robust, imposing privacy constraints without relying solely on encryption or a central node of trust.

Of particular note in this category is the FDCR (Fisher Discrepancy Clustering and Recalibration) framework introduced by Du B. [61]. FDCR gathers client updates based on statistical reliability using the Fisher divergence and refocuses the weight of each cluster during the world aggregation step. This helps downweigh poisoned or unusual updates and maintain diversity during the learning process. This approach was shown to be quite resistant to up to 30 percent poisoning, with the model’s accuracy on benchmarks such as CIFAR-10 remaining at about 65 percent. Since FDCR is model-free and does not rely on a trustworthy dataset, it is particularly appealing in edge conditions where prior knowledge is limited.

Simultaneously, other approaches, such as differential privacy (DP) and secure aggregation protocols, have been widely discussed to address the risks of gradient leakage and inference attacks. DP mechanisms (e.g., DP-SGD, Gaussian noise injection) will restrain the informational extent of every update with the addition of noise, thereby warranting that the impact of any singular example is mathematically bounded [106,133]. However, such techniques come at the cost of accuracy, especially in small-batch or highly non-identical-in-distribution (non-IID) settings [19].

The third category of techniques uses secure aggregation protocols where the server cannot see individual client updates. Alternatively, clients encrypt or obscure their updates in such a manner that only an aggregate (e.g., sum) is disclosed [72]. This prevents an honest but curious server from directly executing gradient inversion on updates to a single client and diminishes the practicality of client-specific profiling. Practical secure aggregation is usually geared towards client dropout tolerance and maintaining a computation that is light enough to run on the edge [134]. There is also homomorphic encryption and variants of secure multi-party computation, which are not deployed extensively due to overhead, unless applied to cross-silo environments or small client groups [135].

Gradient clustering and privacy-preserving techniques have several practical limitations despite their potential. There might be a trade-off when using differential privacy mechanisms in the real-world, non-IID setting, and, due to resource constraints, encryption-based methods may be computationally intensive for devices. Furthermore, clustering-based models would confuse genuine client updates with distinct or minority data distributions. Consequently, the best use of these methods is in a hybrid defense pipeline that combines trust estimation, aggregation filtering, and communication protection.

### 5.3. Anomaly Detection and Client Management

The roles of anomaly identification and client management are essential to protecting federated learning systems from Sybil and free-rider attacks that exploit the open, decentralized participation model. An adversary can create multiple fake identities and amplify their impact on model updates through Sybil attacks [112]. In contrast, in free-rider attacks, malicious participants receive the rewards of the global model without training on any meaningful local data [119]. The effective defenses should thus (i) identify abnormal behavior on updates and patterns of participation, and (ii) control the inclusion, weighting, and incentives of a client in the rounds.

To overcome such threats, researchers have proposed reputation-based systems that assess and document the trustworthiness of the client’s contributions across a sequence of training sessions [19]. In such schemes, clients with continually updated scores on the global learning goal receive a higher reputation score. In contrast, clients with anomalous, noisy, or low-effort updates have their reputations reduced [119]. Reputation-based schemes deter malicious behavior either by refusing to aggregate low-reputation clients or by placing less importance on their contributions in model updates [29].

Trust scoring is a generalization of reputation into a more immediate, quantitative filter that can be run round-by-round. Typical indicators of trust are: (*a*) statistical distance to a dominating update distribution [61], (*b*) gradient proximity to reference benign behavior [132], (*c*) server-side loss when momentarily implementing an update given by a client to the global model [134]. The aggregator can then use adaptive weighting, in which a client’s contribution is directly proportional to its trust [125]. This is particularly applicable in the non-IID case. Instead of the system rejecting clients with legitimate distribution shifts, it can deem only those whose updates are unusual and harmful for validation metrics. Sybils can also be revealed by cluster-aware trust scoring, due to the high correlation of updates (high similarity in direction, timing, and magnitude) created by Sybil identities, which can be aggregated into a suspicious micro-cluster of updates within a round or across rounds.

In addition to anomaly detection, trust-based management systems may incorporate incentive schemes that reward clients based on their trustworthiness and effort, thereby discouraging free-riding [28]. Cryptographic proofs, such as zero-knowledge proofs, can also be used in conjunction with reputation and trust scoring to ensure that clients perform computations demonstrably without revealing raw data. Nevertheless, it remains challenging to design such mechanisms in a federated and edge environment, as devices are heterogeneous, privacy is limited, and continuous monitoring is prohibitively costly [15].

### 5.4. Communication Security and Resilience

The security and resilience of communication are of utmost importance in federated learning and edge computing setups, where adversaries can exploit vulnerabilities in the data transmission channel through eavesdropping, tampering, replay, or denial-of-service attacks [19,102]. To reduce such risks, the latest studies focus on the use of Moving Target Defense (MTD) and redundant transmission strategies as supplementary methods to ensure confidentiality, integrity, and availability [130]. MTD represents an active security model that dynamically alters the system’s attack surface by periodically modifying the communication parameters used—such as IP addresses, ports, encryption keys, or routing paths—thereby making it difficult and expensive for adversaries to attack persistently [136].

MTD can be utilized in federated learning to randomize communication paths between clients and aggregators or to disaggregate protocol stacks, thereby mitigating vulnerabilities to man-in-the-middle attacks, traffic monitoring, and protocol jamming. In a state of constant uncertainty, MTD turns fixed vulnerabilities into real-time opportunities, forcing attackers to evolve on the spot and significantly reducing their chances of success [136]. Simultaneously, redundant transmissions enhance resilience by replicating important updates across multiple communication paths or by scheduling retransmissions to deliver data in the event of packet loss or directed disruption [137].

Even though redundancy increases communication overhead, it enhances fault tolerance and continuity of latency-sensitive operations, especially when used with error-correcting codes and lightweight authentication protocols. Additionally, hybrid approaches that combine MTD with redundancy offer active defense and recovery: even though MTD minimizes the likelihood of successful attacks [138], redundant transmission ensures service continuity during attacks. However, a difficulty in applying these mechanisms to resource-constrained edge settings is balancing security with efficiency, because excessive redundancy can strain bandwidth, and frequent reconfigurations of MTD may cause devices to lose synchronization when they are heterogeneous [23,139].

Overall, there should be adaptive, layered defenses to guarantee the security and resilience of communication in federated learning. Moving Target Defense increases uncertainty for attackers, and duplicate transmissions promote resilience against attacks; other systems also contribute to a space of reliable, attack-tolerant distributed AI systems.

### 5.5. Advanced and Hybrid Frameworks

Current developments in federated learning defense research have led to the development of composite models that combine multiple security and privacy protection mechanisms to become robust against diverse adversarial attacks. The two examples are FDCR (Fisher Discrepancy Clustering and Recalibration) and RFLPA (Robust Federated Learning with Privacy Assurance). FDCR is an architecture that increases robustness by clustering client updates based on their Fisher information discrepancies and reweighting their contributions during aggregation [61]. FDCR identifies and separates obfuscated or low-quality updates that do not conform to the statistical structure of honest clients, thereby protecting against data poisoning, backdoor insertion. In contrast to traditional anomaly detection, FDCR has a recalibration step in which suspicious updates that are identified and excluded from aggregation, trade-offs robustness for fairness, and does not exclude benign clients with non-IID distributions.

In addition, RFLPA offers a hybrid option that combines robustness and privacy and, by overcoming adversarial manipulation, enhances data confidentiality [140]. RFLPA also uses secure aggregation with adaptive weighting to downweight malicious contributions and make gradients resistant to leakage from membership inference or gradient inversion attacks. The framework is notably well aligned with edge environments with resource-constrained devices, particularly because it focuses on low-overhead defenses that remain viable in a practical deployment context.

As demonstrated in both FDCR and RFLPA, there is a tendency to increase the number of layers and multi-purpose defense systems. In contrast, a unilateral approach would not be as practical as adjusting to opponents’ changing tactics [61]. These frameworks combine anomaly detection, resilient aggregations, and privacy-preserving protocols, thereby increasing resistance to integrity and confidentiality attacks. Nevertheless, there are still issues maintaining a trade-off between computational cost and scalability, especially in cross-silo and cross-device deployments.

### 5.6. Decentralized and Cross-Silo FL

Typically, FL traditionally presumes some centralized server that integrates client updates. This architecture is simple, but it introduces a single point of failure, centralizes trust, and can result in a bottleneck when there is large-scale participation by edges. Decentralized and cross-silo FL should help overcome these weaknesses by reassigning control and coordinating learning across organizational scopes and governance limitations.

In FL, the central aggregator is replaced with peer-to-peer collaboration, which is decentralized. Rather than storing data on a server, clients share updates with other peers and implement a consensus-like averaging algorithm. This can enhance tolerance to faults: in the event of the failure of one node, training may resume along alternative paths. Nonetheless, decentralization increases the attack surface, as enemies can attack routing, abuse topology, or isolate a group of clients. Consequently, decentralized FL designs will have to deal with (i) a secure neighbor discovery and authentication, (ii) resilience to partitioning and eclipse-like attacks, as well as with (iii) strong aggregation under partial and local perspectives of the network. Topology-aware robustness has been proposed as a solution to these trade-offs and as a means to ensure convergence under adversarial conditions [32].

Cross-silo FL is typically considered a special case because the parties are fewer, more fixed, and more institutionalized (e.g., hospitals, banks, or government agencies). The threat model has changed: the primary issues now include organizational trust, regulatory compliance, auditability, and accountability, rather than massive churn. This increases the plausibility of stronger security measures (e.g., secure aggregation, stronger authentication, or formal governance controls) but, in turn, increases transparency, reporting, and dispute-resolution requirements when a model’s behavior influences real choices [141].

Frameworks such as HiFGL illustrate how hierarchical coordination can bridge cross-device and cross-silo realities by structuring training into layers (local groups, regional aggregators, global coordination) and modeling relationships as graphs. The technical value is that hierarchy can reduce communication load and allow control at intermediate levels. At the same time, graph modeling captures dependencies among silos or devices when the data is relational in nature [44]. Nevertheless, hierarchy can reintroduce partial centralization, so designs must still mitigate aggregator-targeting risks.

Most federated learning defenses are evaluated under idealized settings that overlook edge constraints. Table 4 summarizes the edge-feasibility trade-offs of major federated learning defense mechanisms with respect to computation, communication, energy cost, and underlying assumptions.

## 6. Defense Mechanisms, Assumptions, and Limitations of Edge Federated Learning

Defense strategies proposed for federated learning (FL) are typically evaluated under implicit mathematical and system assumptions that strongly affect their performance in edge computing. It is important to bring these assumptions to the forefront to determine the feasibility of implementing and measuring a defense approach under real edge-constrained conditions, where data heterogeneity, intermittent connectivity, and limited computational resources are commonplace.

Strong aggregation algorithms such as Krum and Multi-Krum, trimmed mean, and median aggregation are constructed under the assumption that the fraction of bad client participation in each training round is bounded and that benign client updates are concentrated around a relatively small set of statistical modes. These approaches use distance-based or order-statistic filtering to reduce outliers and implicitly assume that non-corrupted gradients are sufficiently similar. However, in edge FL, this assumption is invalid because non-IID data distributions and heterogeneous device behavior are natural; thus, legitimate updates fail to converge substantially to the global mean. As demonstrated by previous research, it may lead to benign updates being falsely filtered or malicious updates being stored, thereby degrading convergence and model utility [13,64].

Defenses based on differential privacy (DP) provide formal privacy guarantees that bound the sensitivity of updated models and use controlled noise in either local training or aggregation. These guarantees require relatively large local datasets, stable participation across training rounds, and well-tuned privacy budgets. In edge environments, however, clients often have small, skewed datasets and are involved sporadically, which amplifies the effect of injected noise and quickly depletes the privacy budget. In fact, empirical results indicate that, in this context, non-IID data distributions can be highly effective at reducing model accuracy and convergence stability when used with DP mechanisms [15,25]. Consequently, DP remains theoretically sound, but its utility in edge FL is constrained by stringent resource and data constraints.

Secure aggregation schemes, such as multi-party computation protocols and masked summation protocols, rely on effective synchronization among all involved clients and the completion of multiple-round communication protocols [82,133]. These assumptions are often violated in edge-based systems due to intermittent connectivity, device churn, and energy constraints. Secure aggregation can stall or require expensive recovery protocols when clients fail or drop out, thereby adding latency and communication overhead and typically increasing the protocol’s cost [129]. Although this is possible in cross-silo FL or infrastructure-based edge deployments, the mathematical and operational modeling of this type of defense does not scale uniformly to large-scale, cross-device edge deployments.

There are more assumptions about the statistical separability of benign and malicious updates in trust-based aggregation and anomaly detection techniques. The FLTrust, clustering-based anomaly detection, and contribution auditing techniques are based on reference behaviors, validation signals, or client historical statistics, and assign trust scores or weights. Subconsciously, such techniques are based on the idea that bad behavior is always inconsistent with trusted updates. Benign clients can become highly variable in highly heterogeneous edge environments; however, when sensing contexts, hardware capabilities, or data distributions differ, the risk of false positives and biased client suppression is high. This compromises both strength and equity, especially in prolonged deployments.

## 7. Analysis of Related Studies and Discussion

To gain a clearer understanding of the history and complications of federated learning (FL) research, it would be beneficial to critically divide the literature into three main areas of study: system architectures, attack strategies, and defense mechanisms. In addition to providing an improved perspective for examining the various studies, this organized review also enables a better understanding of any gaps and overlaps within the field.

Firstly, FL systems, from an architectural perspective, are implemented with either a centralized or a decentralized coordination model. Centralized systems rely on a single server to manage both training and aggregation across clients. This type of model, which uses the FedAvg algorithm, as exemplified by McMahan [55], is conceptually straightforward and computationally efficient; however, it has serious drawbacks, including vulnerability to single-point failures, scalability issues, and communication bottlenecks when handling large datasets. Conversely, the decentralized, cross-silo-attribute structures, more thoroughly discussed in papers by Yuan et al. [32] and further surveyed by Hallaji et al. [35] Divide the coordinator tasks among many clients. Such systems enhance fault tolerance, require less trust, and are designed to fit industrial IoT and deployment in multi-organizational projects. They, however, introduce new issues related to synchronization, security, and network overhead.

The second broad category encompasses the scope of opposition challenges that undermine FL’s integrity. The FI attacks are usually described based on their methods and targets. For example, poisoning attacks aim to undermine model behavior by skewing clients’ updates, either subtly, as in backdoor attacks or without any guise, as in label flipping [9]. Zhu et al. [10] provided an example of a gradient leakage attack that exploits a shared update to recover sensitive client data. Other identified threats include model inversion, membership inference [7], and Byzantine behavior [14], in which malicious clients introduce incorrect updates, making the training unstable.

Lastly, the third main research category is held by the defense mechanisms. The earlier methods focus on strong aggregation, such as Krum [14], which selects the updates closest to the statistical mean to suppress outliers. Newer frameworks, including FLTrust [132] and FLIP [48] introduce concepts such as trust modeling, adversarial hardening, and discrepancy-based clustering to enhance resilience against targeted attacks. These mechanisms differ considerably in terms of computational complexity, strength, and capabilities in the context of edge computing.

Since adversarial threats in federated learning (FL) are widespread, it is imperative to understand the full range of defense measures to counter them. Every kind of attack—those targeting privacy, integrity, aggregation, and communication—requires specific countermeasures that account for the limitations of edge devices, the diverse nature of data, and the dynamic participation of clients. Table 5 summarizes the mapping of the significant attack classes covered in Section 4 to the most common defense mechanisms to mitigate them. The table also highlights the targeted nature of each attack, the main sources cited, and the restrictions on the available preventive measures. Such a systematic perspective enables both researchers and system architects to evaluate the most suitable defense methods for their respective threat models and deployment contexts.

Although some defense strategies, such as differential privacy, robust aggregation (e.g., Krum, RFA), and client trust scoring, have shown promise in addressing a wide range of attacks, they cannot be considered the most universal mechanisms, as each method has its drawbacks. For example, privacy-preserving mechanisms tend to reduce model utility, and robust aggregation mechanisms might perform poorly in non-IID scenarios or when the ratio of adversaries exceeds the assumed attacker rate. Furthermore, some existing defense strategies, such as homomorphic encryption and secure multiparty computation (SMC), despite being theoretically successful, are highly computationally and communication-expensive and thus not applicable in lightweight edge implementations.

This mapping highlights one primary concern in the present-day body of FL security research: the absence of dynamic, hybrid defense paradigms capable of adapting to changing threats across multiple dimensions (data, communication, aggregation). It also highlights that, in practice, a trade-off among robustness, scalability, and performance is inevitable. Future research in this field should focus on integrating these countermeasures into modular, context-aware defense pipelines that support heterogeneous, continuously changing FL deployment environments.

Modern literature contains severe inconsistencies regarding methodology, assessment, and practical application to reality. First, the vast majority of defense mechanisms have been considered in constrained scenarios with little or no consideration of heterogeneous edge devices or distributed planning alternatives [1,41]. For example, Krum and Multi-Krum are barely tested beyond simulated IID datasets, making them inapplicable to edge-deployment scenarios. Moreover, although many studies used standard baselines, most of them lack cross-model evaluation of threats or cross-FL (under FL: device vs. silo). For example, although FLTrust has good resilience against targeted poisoning [13], little is known about its resilience against Sybil attacks. Similarly, HiFGL also presents a new view of hierarchy [44], although it was not compared with simpler models of the same network [97]. Second, communication costs/energy usage are not included in most assessments, yet these are among the most critical aspects of edge computing [23]. Lightweight defense schemes such as RFLPA [140] and FDCR [61] exist as frameworks, although their suitability under bandwidth constraints has not been rigorously assessed. Third, in the literature, there is limited exploration of hybrid defenses that incorporate multiple strategies (e.g., adversarial detection, robust aggregation, and communication-efficient updates). This leaves a hole in the process of designing resilient, layered FL systems suitable for deployment. Lastly, publicly accessible benchmarks with realistically simulated edge settings (e.g., mobile clients and intermittent connectivity, adversarial distributions) are needed. The lack of uniform assessment scales also poses a vulnerability to reproducibility and comparability.

## 8. Emerging Topics in Edge Federated Learning

Recent developments in federated learning (FL) for edge computing are no longer centered on the classical FedAvg-style perspective but are gradually shifting toward an emphasis on heterogeneity, architectural hybrids, large-model training, resource awareness, and realistic frameworks. In this section, we identify the research directions that have gained prominence since 2022 and those that are particularly applicable to real-world edge deployments.

- *Personalized FL of heterogeneous edge devices*

One of the main advancements in recent edge FL is the shift from a single global model to a personalized approach to address statistical and system heterogeneity. Personalized FL is starting to be viewed as increasingly needed in edge and IoT environments where data distributions of clients vary significantly, sensing contexts vary significantly, and hardware features vary significantly. Health monitoring is one area of cloud-edge personalization, where personalization has been shown to enhance utility in a heterogeneous home setting [3]. Hierarchical personalization across large mobile network edges further illustrates how personalization can be integrated with multi-tier edge architectures to balance latency and performance [68]. In a broader sense, emerging research on personalized FL emphasizes the formalization of personalization objectives and strategies as a sign of this trend’s maturity, particularly in moving beyond the simplicity of single-model-fits-all deployments of FL [56]. The current hierarchical client-edge-cloud personalization also emphasizes system designs tailored to mobile edge environments and a variety of mobile devices [68].

- *Split learning and hybrid FL-SL models*

A second development is split learning (SL) and hybrid FL-SL architectures, which reduce on-device computation and reduce exposure to raw features, and scale better under edge conditions [31]. Specifically, split-federated learning models, in which federated coordination is integrated with partitioned model training, have been introduced in large-scale recommendation systems in edge-cloud pipelines, where hybridization can also trade local compute against communication and edge/cloud execution [42]. This body of work motivates revisiting the threat surface, as hybrid training introduces new points of attack (e.g., intermediate activations, cut-layer communication) and new feasibility trade-offs for defenses compared to standard FL.

- *Edge FL with larger and foundation-scale models*

Recent efforts are also moving toward training larger models using end-to-end training [81]. Likewise, the nature of domain-based surveys, including federated learning in computer vision, signifies the expanding scale and complexity of edge FL workloads, with model size, representation leakage, and training stability becoming more of a concern [69]. These advances inspire a re-evaluation of privacy leakage (e.g., representation-based leakage) and integrity attacks (e.g., targeted backdoors) in larger-capacity models and feature spaces.

- *Efficiency-conscious orchestration/resource/cost-conscious FL*

Resource- and cost-conscious FL focuses on optimizing client selection, participation policies, and aggregation schedules, subject to communication, energy, and latency constraints. It has been suggested that adaptive client sampling can address statistical and system heterogeneity simultaneously, with sampling policies significantly affecting performance and robustness in heterogeneous settings [16]. A design dimension that has received central treatment is communication efficiency, and surveys have been used to trade off compression, fewer rounds, and convergence behavior under limited bandwidth [46]. Practical, robust, and communication-efficient FL on non-IID data further highlights the non-trivial trade-offs between robustness and efficiency at the edge [46]. In general, edge deployment and orchestration efficiency has become a well-studied systems concept, offering algorithmic underpinnings that can be adapted in FL scheduling and placement decision-making [77]. Most recent work on IoT has also examined the design of energy-efficient FL for IoT, suggesting continued focus on quantifying and minimizing energy/overhead in practical deployments [89]. Collectively, these efforts strengthen the manuscript’s feasibility claims by grounding them in actual system cost dimensions rather than qualitative assertions.

- *Frameworks and architectures of FL at the system level*

Lastly, the latest literature highlights system architecture and frameworks that implement FL at the edge, such as decentralized/hierarchical coordination, use of blockchain-based trust, and standard architectural designs. Decentralized FL surveys provide current perspectives on architectures, trends, and practical system design considerations that inform security proposals (e.g., trust limits, peer-to-peer organization) [29]. Recurring components are further explained by architectural patterns in FL system design and support security reasoning and defense placement in end-to-end systems, thereby making them more systematic [51]. Integrated FL with blockchain is also considered as a trust, auditability, and decentralized coordination mechanism in edge environments, and additional security considerations and overhead trade-offs [95]. Broadly speaking, the industrial and IoT-based discourse on decentralized FL highlights the real deployment issues and opportunities that shape security and privacy requirements at the edge [1]. These system-level views encourage consideration of defenses not only in terms of algorithmic robustness but also in terms of deployability within real architectural stacks.

In general, FL has expanded beyond federated optimization at the edge to encompass personalization, hybrid training paradigms, multi-model collaboration, resource-aware orchestration, and end-to-end orchestration. This development has a direct influence on the security and privacy landscape: personalization and heterogeneity make it harder to detect anomalies and to make strong aggregation assumptions; hybrid FL-SL creates new leakage and tampering points; larger models increase representational leakage and backdoor risks; and system structures create new trust and governance considerations and vulnerabilities.

## 9. Open Challenges

The recent literature confirms that Federated Learning (FL) has become a central paradigm in privacy-preserving machine learning, enabling joint model training without requiring centralized access to sensitive information. In various fields, such as healthcare, finance, edge computing, and Industrial IoT, the use of FL has been demonstrated to be highly beneficial for ensuring user privacy while maintaining strong model performance. Using healthcare as an example, in FL, a hospital and a clinic can collaboratively develop predictive models using patient data without disclosing personal health information, which is essential given regulatory frameworks such as GDPR and HIPAA. Likewise, in Industrial IoT, FL can enable distributed devices to contribute to predictive maintenance and anomaly detection without sending proprietary operational data to central servers, minimizing security risks and communication costs.

The algorithms used in the reviewed studies exhibit subtle variations, depending on data distribution and system heterogeneity. FedAvg is a simple option with good performance under IID (Independent and Identically Distributed) data, but it underperforms under non-IID conditions when client heterogeneity is high. In this case, algorithms such as FedProx and FedAvg have been shown to ensure convergence stability by adding regularization terms that alleviate client drift.

By combining Differential Privacy (DP) and Secure Aggregation (SA), it is demonstrated that FL systems are resistant to privacy inference attacks and to malicious clients that may attempt to invert the models or inject gradient leakage. However, introducing these privacy-sensitive measures typically creates a trade-off between privacy and utility, as noise injection and encryption overheads may slow convergence and reduce accuracy. This observation also underscores the importance of adaptive algorithmic solutions that balance privacy, security, and model performance in practice.

In FL, security threats remain a significant concern, particularly poisoning attacks, backdoor insertion, and evasion/avoidance strategies that exploit flaws in collaborative learning. The analyzed literature indicates that although defenses such as DP, SA, and Homomorphic Encryption (HE) can mitigate most risks, they remain incomplete against dynamically and adaptively evolving attacks that evolve across multiple training rounds. In addition, edge devices and industrial sensors introduce further complexities, including limited computational capabilities, intermittent connectivity, and heterogeneous hardware, which directly impact both model and defensive reliability. The other lesson is that there is a dearth of universal assessment metrics: many studies report accuracy, F1-score, or loss functions separately, making it challenging to cross-test them. Consistent metrics and benchmarks are thus essential for reliable evaluations of FL systems across various applications and datasets, including structured, image, text, and audio.

In addition to technical factors, deployment factors indicate that implementing FL requires not only algorithmic efficiency but also organizational preparedness, data governance, and resilient infrastructure. However, collaborative systems often require the smooth coordination of stakeholders with varying levels of technical skill, which is why modular, scalable FL architectures are essential. The literature also indicates that studies involving multimodal FL, integrating image, text, and signal information, remain scarce. This is a promising area for future efforts to expand the scope and range of FL in complex real-life situations.

Although federated learning security is advancing rapidly, several open issues and research gaps remain that hinder the implementation of robust, practical systems.

- ***The emergence of adaptive attacks***, in which adversaries continue to develop tactics that align with current defenses. The majority of proposed solutions are based on static threat models; however, attackers may dynamically optimize the magnitude of poisoning, adjust gradient inversion parameters [8], or orchestrate Sybil identities to evade both anomaly detection and privacy-preserving solutions [112].
- ***The immature nature of hybrid defenses***, which combine anomaly detection, robust aggregation, differential privacy, and cryptographic protocols. Although models such as FDCR and RFLPA are promising steps towards multi-layered resilience, they typically add overhead and are not systematically integrated. The challenge of developing lightweight, adaptable frameworks to protect against concurrent threats to integrity, confidentiality, and availability remains open.
- ***Scaling security mechanisms***, as most are run in small-scale environments or simulations. In contrast, real systems have thousands of heterogeneous clients with different computational, storage, and energy constraints. Ensuring the efficiency of defenses such as secure aggregation, Byzantine-robust aggregation, and moving-target defense in cross-device, cross-silo, and IoT-scale systems remains a challenge.
- ***The lack of real-world metrics*** to assess the security of federated learning. Simplified datasets, such as MNIST or CIFAR, are frequently used in current research and do not accurately reflect the multifacetedness, heterogeneity, and complexity of problems like healthcare, finance, and autonomous systems. In the absence of standard benchmarks, defenses cannot be easily compared, evaluated for resilience against adaptive threats, or demonstrated to scale in real-world deployments.

In addition to the previous security-related gaps, some additional gaps are related to the context of FL itself and may have implications for security issues:

- A few studies exist on FL with multi-modal data (text, audio, and industrial sensor streams).
- Lack of proper developmental mechanisms to tackle the issue of heterogeneity and scalability of the system as deployed into the real world.

Overall, addressing the above gaps, incorporating adaptive attacks, implementing hybrid defenses, ensuring scalability, and conducting real-world benchmarking will be crucial to ensuring that federated learning is no longer confined to controlled laboratory environments and that its results are effectively translated into trustworthy, large-scale applications. It is necessary to overcome these challenges through interdisciplinary cooperation across machine learning, cybersecurity, and distributed systems research, as well as through collaboration with industry to develop realistic testbeds and evaluation procedures.

## 10. Future Research Directions

Moving forward, a set of research directions is key to improving the security and resilience of federated learning and edge computing.

- The top priority is ***achieving scalability for IoT and 5G networks***, which must integrate thousands of distinct device types with varying computational, bandwidth, and energy capabilities safely and securely. Current protection mechanisms, such as secure aggregation and Byzantine-robust protocols, do not scale well in these environments and instead require simple yet robust operations in large-scale cross-device applications.
- The other critical direction is ***creating adaptive, hybrid defense architectures*** that can respond to changes in adversarial strategies. This is because attackers continually develop new methods of poisoning, interference, and free-riding, so a static defense will not be sufficient. New architectures should integrate anomaly detection, differential privacy, cryptographic schemes, and reputation-based systems into multi-layered pipelines that are adaptive and dynamically respond to threats in real time.
- In addition to ***flexibility***, the community should seek ***energy-conscious, lightweight security solutions,*** as most edge devices face severe resource constraints. It will be necessary to design mitigation mechanisms to reduce cryptographic overhead, communication rounds, and energy consumption, thereby enabling long-term deployment in IoT and mobile ecosystems.
- ***The standardization of benchmarks and metrics*** is also an urgent issue, as the existing analysis is based on a small data set and simulation conditions. Standardized, application-specific testbeds, such as those for healthcare, transport, and industrial IoT, can be used to compare defenses uniformly, ensure reproducibility, and adapt defenses to safety-related applications.
- Lastly, researchers should expand the list of hazards and study ***more contemporary attack models***, including side-channel attacks that exploit hardware leakage, timing, or energy consumption to reveal sensitive information from edge nodes. These understudied vectors are realistic threats to decentralized systems and require new countermeasures, including hardware-level security and federated protocols.

In short, the next stage of federated learning security research will be characterized by further progress in scalability, adaptability, efficiency, benchmarking, and threat exploration. To overcome these issues, interdisciplinary teams spanning machine learning, cybersecurity, and networking, as well as increased industry interactions, are required to create real-world, deployable solutions.

## 11. Conclusions

Federated learning is a new trend in AI development that enables privacy-aware collaborative learning across distributed data sources. The literature review suggests that FL is particularly applicable to sensitive sectors, such as healthcare, finance, and industrial IoT, where centralized data collection is expensive and stringent privacy laws are in place. The efficacy of algorithmic options, e.g., FedAvg, FedProx, and DP-FL, is highly contingent on the data distribution and privacy conditions and therefore may degrade under non-IID settings. Security and privacy mechanisms, such as differential privacy, secure aggregation, and homomorphic encryption, should be incorporated to protect FL systems against the most common attacks. Still, these features frequently involve significant performance trade-offs.

FL has other practical implications beyond privacy protection. FL can be used to support distributed intelligence with low latency in edge computing and IoT systems, where it updates local models with local data and decouples from higher-rate communications.

The following steps in this research aim to enhance adaptive, resilient FL frameworks that strike the optimal balance between privacy, security, and model utility across various domains. Also, studying hybrid methods that combine FL with transfer learning or physics-informed neural networks may dramatically increase usage across non-eBay sectors and environments with high-stakes decisions and domain-specific information. The development of standardized evaluation procedures with detailed metrics for accuracy, robustness, and privacy leakage will be essential to enable meaningful benchmarking and accelerate the adoption of FL. In this context, FL is a crucial step towards AI-based technologies and the prospect of safe, cooperative, and intelligent systems. However, to realize its full potential, it is essential to continue research to enhance the security, privacy, and functionality of heterogeneous and real-world systems.

Overall, the experience with federated learning and edge security highlights a twofold truth: the paradigm has the potential to enable collaboration with intelligence without compromising data locality, which is inherent to its nature. These difficulties cannot be resolved solely through technical innovation, which adaptive, lightweight, and scalable defense architectures can provide, but also through interdisciplinary teamwork and long-term research to translate academic progress into working, deployable products. Further investigation of novel threat models and the development of standardized benchmarks will play a key role in realizing the potential of federated learning and edge computing as trustworthy and secure foundations for next-generation distributed AI.

## Figures and Tables

**Table 1:** Overview of previous survey studies on federated learning and edge computing.

Ref.	Title	Focus	Findings/Contributions	Year	FL	EC	Attacks	Defense
[17]	Federated machine learning: Concept and applications	FL concepts & applications	Defines FL, motivates use-cases and core workflow	2019	✓			
[18]	A Survey of Federated Learning for Mobile Edge Networks	FL in mobile edge networks	Surveys FL fundamentals, MEC integration, challenges, and security/privacy issues	2020	✓	✓	◐	◐
[12]	A survey on security and privacy of federated learning	Security + privacy	Broad mapping of threats and defenses across FL	2021	✓		✓	✓
[19]	Advances and open problems in federated learning	Open problems	Consolidates advances + research gaps	2021	✓	◐		
[20]	A Survey on Federated Learning	General FL survey	Comprehensive FL survey: taxonomy, privacy, heterogeneity, applications	2021	✓	◐	◐	◐
[21]	Federated Learning for Internet of Things: A Comprehensive Survey	FL in IoT environments	Surveys FL in IoT, including architectures, applications, and security/privacy challenges.	2021	✓	◐	◐	◐
[22]	A survey of federated learning for edge computing: Research problems and solutions	FL in edge environments	Survey of FL challenges, security, and solutions	2021	✓	✓	✓	✓
[23]	Federated learning for edge computing: A survey	Edge FL	Reviews edge-FL architectures & challenges	2022	✓	✓		
[24]	Differential Privacy for Deep and Federated Learning: A Survey	DP for FL	Reviews DP variants for deep/FL and implications	2022	✓		✓	✓
[25]	Federated learning in edge computing: A systematic survey	Edge FL	Systematic mapping of edge-FL methods	2022	✓	✓		
[26]	Privacy and Security in Federated Learning: A Survey	Privacy and security in FL	Surveys security and privacy threats in FL and reviews corresponding defense mechanisms	2022	✓	◐	✓	✓
[27]	Combined Federated and Split Learning in Edge Computing for Ubiquitous Intelligence in Internet of Things: State-of-the-Art and Future Directions	FL, SL, in edge-based IoT	Survey on hybrid FL–SL frameworks, architectures, and challenges	2022	✓	✓	◐	◐
[28]	A survey on federated learning: challenges and applications	FL challenges/apps	Summarizes challenges and applications	2023	✓	◐	✓	✓
[29]	Decentralized Federated Learning: Fundamentals, State of the Art, Frameworks, Trends, and Challenges	Decentralized FL	State-of-the-art and trends for decentralized FL	2023	✓	◐		
[30]	A Survey on Securing Federated Learning: Analysis of Applications, Attacks, Challenges, and Trends	Securing FL	Analysis of applications, attacks, defenses	2023	✓	◐	✓	✓
[31]	Combining Federated Learning and Edge Computing Toward Ubiquitous Intelligence in 6G Network: Challenges, Recent Advances, and Future Directions	FL–Edge integration for 6G	Survey of FL–edge architectures, challenges, and future directions	2023	✓	✓	◐	◐
[15]	Survey: Federated Learning Data Security and Privacy-Preserving in Edge-Internet of Things	Edge-IoT privacy/security	Surveys FL privacy/security in edge-IoT constraints	2024	✓	✓	✓	✓
[32]	Decentralized federated learning: A survey and perspective	Decentralized FL	Reviews decentralized FL + open challenges	2024	✓	◐	◐	
[33]	Membership Inference Attacks and Defenses in FL: A Survey	MIA	Surveys MIA + defenses in FL	2024	✓		✓	✓
[34]	Anomaly detection and defense techniques in FL: a comprehensive review	Anomaly detection	Surveys anomaly detection defenses	2024	✓		✓	✓
[35]	Decentralized Federated Learning: A Survey on Security and Privacy	Security/privacy in decentralized FL	Security/privacy survey for decentralized FL	2024	✓	◐	✓	✓
[36]	The Impact of Adversarial Attacks on Federated Learning: A Survey	Adversarial attacks	Surveys adversarial attacks and impacts	2024	✓		✓	✓
[37]	Topology-aware Federated Learning in Edge Computing: A Comprehensive Survey	Topology-aware FL in edge computing	Reviews topologies, challenges, and security issues	2024	✓	✓	◐	◐
[38]	A survey of security threats in federated learning	FL security threats	Survey of threats and mitigation themes	2025	✓	◐	✓	✓
[13]	SoK: Benchmarking Poisoning Attacks and Defenses in Federated Learning	Defense benchmarking	Benchmarks defenses under common settings	2025	✓		✓	✓
[39]	The Federation Strikes Back: A Survey of Federated Learning Privacy Attacks, Defenses, Applications, and Policy Landscape	Privacy attacks/defenses & policy	Surveys privacy attacks/defenses + policy landscape	2025	✓	◐	✓	✓
This Work	Federated Learning in Edge Computing: Vulnerabilities, Attacks, and Defenses: A Survey.	Adversarial threats and defense feasibility in edge FL	Surveys vulnerabilities, attacks, and defenses in edge FL.	2026	✓	✓	✓	✓

Legend: ✓ explicit coverage; ◐ partial coverage.

**Table 2:** Federated learning aggregation algorithms.

Algorithm	Key Idea	Strengths	Limitations	Suitable Scenarios
FedAvg	Weighted averaging of client updates [20]	Simple, widely adopted, and low communication overhead.	Poor with non-IID data, slow convergence.	Homogeneous clients, balanced data.
FedProx	Adds proximal term to local objective [61].	Handles data heterogeneity better than FedAvg.	Higher computation cost.	Non-IID client data, healthcare, IoT.
FedNova	Normalizes updates to address client imbalance [20]	Prevents bias from fast/slow clients.	More complex implementation.	Edge settings with mixed device speeds.
Scaffold	Uses control variates to reduce client drift [12].	Improves convergence with non-IID.	Requires extra storage/communication [62].	Large-scale heterogeneous systems.
MOCHA	Multi-task FL optimizes per-client models [58,63].	Personalized performance.	Higher overhead, complex training.	Personalized healthcare, finance.

**Table 3:** Applications of the Federated Learning system in edge computing.

Domain	Example Application	Benefits of FL	Challenges Observed
*Smart Cities* [25,83].	Traffic control & surveillance with edge cameras	Privacy-preserving video analytics; reduced bandwidth	Non-IID video data; device heterogeneity
Autonomous Vehicles [2,41].	Collaborative driving models across cars	Low-latency decision-making; shared learning without raw data	Scalability; data and system heterogeneity potential adversarial attack
Healthcare IoT [6].	Wearables for patient monitoring (heart rate, blood pressure)	Preserves patient privacy; enables personalized medicine	Gradient leakage risks; device energy limits
Energy Systems [11,85].	Smart grid load forecasting using smart meters	Improved prediction accuracy; privacy-preserving data sharing	Communication bottlenecks; limited computation at edge
Home/Residential IoT [3,86].	In-home health monitoring (e.g., FedHome)	Personalized models; data sovereignty	Device diversity; intermittent connectivity

**Table 4:** Edge-Feasibility Analysis of Major Federated Learning Defense Mechanisms.

Defense Category	Representative Methods	Device-Side Compute Cost	Communication Overhead	Energy Impact	Key Assumptions	Edge Feasibility Summary
Robust Aggregation	Krum, Multi-Krum, Trimmed Mean	Low–Moderate	Low	Low	Bounded attacker fraction; IID-like gradients	Feasible at edge servers, but unreliable under strong non-IID data common in edge FL
Trust-Based Aggregation	FLTrust, FoolsGold	Low (device), Moderate (server)	Low–Moderate	Low	Trusted reference dataset or stable similarity patterns	Practical at edge servers; difficult in fully decentralized or trust-free settings
Differential Privacy	DP-SGD, Gaussian Noise Injection	Moderate–High	Low	Moderate–High	Privacy budget tuning; tolerance to accuracy loss	Feasible only with careful tuning; accuracy degradation amplified on small edge datasets
Secure Aggregation	MPC-based SA, Masked Summation	Low–Moderate	High	Moderate	Synchronized rounds; reliable connectivity	Challenging under intermittent connectivity; more suitable for cross-silo edge FL
Anomaly Detection	Clustering-based, Loss-based filtering	Moderate	Moderate	Moderate	Availability of validation signals or statistics	Feasible at edge servers; false positives likely under heterogeneous data
Backdoor-Specific Defenses	FLIP, FDCR	Moderate	Moderate	Moderate	Trigger assumptions; attack persistence	Promising but limited against adaptive or multi-round backdoors
Cryptographic Defenses	Homomorphic Encryption, SMC	Very High	Very High	High	Strong compute and bandwidth availability	Generally infeasible for resource-constrained edge devices
Communication-Level Defenses	MTD, Redundant Routing	Low	Moderate–High	Low–Moderate	Network coordination and synchronization	Feasible at edge infrastructure level; limited at end devices

**Legend**: Low/Moderate/High indicates relative overhead compared to standard FedAvg-based training.

**Table 5:** Mapping of attacks and defenses in federated learning literature.

Attack Type	Targeted Aspect	Attack Mechanism	Key Reference	Dataset	Defense Method	Limitations (Study-Specific)
Gradient inversion	Privacy	Adversary reconstructs inputs from shared gradients/updates	[10]	MNIST, CIFAR-100, SVHN, LFW	Secure aggregation; Differential privacy; Gradient compression.	Defenses require strong noise or high pruning ratios, which may reduce accuracy; cryptographic defenses limited.
Privacy Inference	Privacy	Attacker infers sensitive features from shared representations/updates	[91]	LFW, FaceScrub, PIPA, CSI, FourSquare, check-ins dataset, Yelp-health reviews dataset, Yelp-author reviews dataset.	No effective defense demonstrated; **secure aggregation**, sharing fewer gradients; **Dimensionality reduction**; **Dropout**; **Differential Privacy** discussed.	Secure aggregation does not prevent participant-side inference; participant-level DP is impractical under non-IID data.
Membership inference	Privacy	MIA-BAD: Batch-wise loss/confidence–based membership inference	[115]	MNIST, Fashion-MNIST, CIFAR-10, CIFAR-100	**Federated Learning** (more clients reduce attack accuracy)	FL mitigates but does not prevent MIA; effectiveness depends on client count.
Privacy inference threats	Privacy	Adversary infers node features, neighbors, or subgraph structure from message passing and aggregation.	[44]	Cora, CiteSeer, PubMed	**HiFGL**: hierarchical architecture (device-silo-server).	Communication overhead; privacy-only focus.
Byzantine Model poisoning	Integrity	Malicious clients send arbitrary gradients to derail convergence	[14]	Spambase dataset, MNIST	Use **Byzantine-robust aggregation** (e.g., Krum-like selection of consistent gradients)	Robust aggregation assumes a bounded attacker fraction and degrades under high gradient variance in non-IID settings.
Adaptive model poisoning	Integrity	Attacker adapts updates to bypass simple robust filters	[107]	*diverse image datasets*	**Adaptive aggregation defenses** that adjust weighting/filtering based on observed update behavior (FEDADAGRAD, FEDYOGI, FEDADAM)	Adaptive defenses can be bypassed if attackers infer weighting rules; non-IID clients may be falsely penalized.
Poisoning Attacks	Integrity	Malicious clients manipulate local updates or labels to degrade convergence or induce targeted misclassification.	[110]	MNIST	FedGuard: **Selective aggregation** of client updates based on performance evaluation on synthetic validation data.	Effectiveness is drop when many malicious clients/decoders form a large fraction; may require server learning rate tuning.
Model Poisoning	Integrity	Attackers poison updates exploiting resource constraints	[82]	MNIST, KDDCup, Amazon IID & non-IID	**Robust FL design with privacy-preserving components** against poisoning	Added robustness and privacy controls increase system overhead and may not scale to large cross-device edge deployments.
Model Poisoning	Integrity	Malicious clients manipulate gradients or flip labels to bias training	[140]	MNIST, Fashion-MNIST, CIFAR-10	RFLPA—Hybrid Defense: Secure aggregation, FLTrust-based cosine-similarity robust aggregation	Requires clean server root dataset; cryptographic overhead; slightly lower accuracy without attacks
Backdoor Attack	Integrity	Attacker implants trigger-specific behavior while preserving global accuracy; Model Replacement; Constrain-and-Scale; Single-shot capability.	[9]	CIFAR-10, Reddit Corpus	No effective defense within standard FL; **anomaly detection**; **Byzantine-robust aggregation**; and **participant-level DP** are analyzed.	Existing defenses rely on strong assumptions or incur accuracy loss and are often ineffective in standard FL settings.
Backdoor Attack	Integrity	Backdoor signal concentrated in specific NN layers/blocks	[109]	Grid-level energy dataset	LBAA-FedAVG (layer-wise anomaly-aware aggregation)	Effective only under low attacker ratios; +19% training time.
Runtime backdoor activation	Integrity	Detect backdoor by analyzing representational shifts/dissimilarity at runtime	[105]	MNIST, Fashion-MNIST, (FMNIST), CIFAR-10	Representational dissimilarity analysis; LOF	Requires representative probing inputs and continuous monitoring; sensitive to distribution shift and false positives.
Backdoor Attack	Integrity	Malicious clients inject trigger via poisoned local training (single-shot & continuous)	[48]	MNIST, Fashion-MNIST, CIFAR-10	**FLIP**: trigger inversion + adversarial training + confidence thresholding	Depends on trigger recovery quality; slight accuracy drop; static backdoors only
Backdoor attack	Integrity	Clients poison local data with trigger patterns and target labels to manipulate global model	[61]	CIFAR-10, MNIST, Fashion-MNIST	**FDCR**—Hybrid Defense	Cannot remove already embedded backdoors; mitigation limited to aggregation stage
Sybil attack	Integrity + fairness	One adversary creates many clients to dominate aggregation	[123]	MNIST, CIFAR-10	**FoolsGold**: Similarity-based Sybil mitigation (penalize highly correlated clients)	Similar benign clients may be penalized under non-IID data, affecting fairness and contribution balance.
Sybil-based poisoning	Integrity	Attacker amplifies targeted poisoning via multiple Sybil clients.	[124]	MNIST, Fashion-MNIST, CIFAR-10 **(under IID and non-IID Dirichlet distributions)**	None	High computation cost; Limited scalability Degrades with non-IID data; Untested against defenses.
Free-rider attack	Incentives + integrity	Fake gradient generation without local training random sampling, subtracting global models, delta + Gaussian noise	[119]	MNIST	Anomaly detection on model updates (STD-DAGMM).	Statistical detection may misclassify honest non-IID or low-resource clients and adds monitoring overhead.
Free-rider Attack	Incentives + integrity	Free-rider produces low-entropy/replayed gradients	[122]	MNIST, CIFAR	FRAD based on contribution modeling; DAGMM-based free-rider detection	Energy and bandwidth constraints limit continuous detection; features can be spoofed under unstable participation.
DoS/DDoS Attack	Availability + Resource exhaustion	Attack disrupts aggregator/clients, blocks rounds, increases dropouts	[98]	UNSW-NB15	FLEAM: FL-empowered DDoS mitigation using IMA-GRU across collaborators on edge.	Defense introduces additional overhead and its scalability against large-scale or multi-vector attacks is unclear.
Communication attack	Availability integrity confidentiality	Attackers exploit dynamic topology, spoofing, surveillance	[130]	MNIST	**Moving Target Defense** (rotate paths/parameters); **Encryption**	Frequent reconfiguration increases synchronization overhead and may degrade performance under network instability.
Asynchronous Byzantine Attack	Integrity	Attack exploits stale/async updates to bypass robust filters	[142]	MNIST, FMNIST, HAR, Colorectal MNIST, CIFAR-10	**AFLGuard**: Byzantine-robust asynchronous FL with guarded aggregation.	Robustness relies on delay assumptions and may reduce throughput or slow convergence under high churn.
Trust-based Byzantine Attack	Integrity	Malicious clients drift away from trusted behavior	[132]	MNIST-0.1, MNIST-0.5, Fashion-MNIST, CIFAR-10, HAR, CH-MNIST	**FLTrust:** Trust bootstrapping using a small, trusted dataset to evaluate client updates	Requires a representative trusted dataset; bias and scalability issues arise if trust data mismatches global distribution.

**Legend:** DP: Differential Privacy; MIA: Membership Inference Attack.

## Data Availability

Not applicable.
